# Prevalence of cardiac amyloidosis in screening studies: a systematic review and meta-analysis

**DOI:** 10.1093/eschf/xvag172

**Published:** 2026-06-22

**Authors:** Alberto Aimo, Vincenzo Castiglione, Giorgia Panichella, Marianna Fontana, Michele Emdin, Giuseppe Vergaro

**Affiliations:** Health Sciences Interdisciplinary Center, Scuola Superiore Sant’Anna, piazza Martiri della Libertà 33, 56127 Pisa, Italy; Cardiology Division, Fondazione Toscana Gabriele Monasterio, via Moruzzi 1, 56124 Pisa, Italy; Health Sciences Interdisciplinary Center, Scuola Superiore Sant’Anna, piazza Martiri della Libertà 33, 56127 Pisa, Italy; Cardiology Division, Fondazione Toscana Gabriele Monasterio, via Moruzzi 1, 56124 Pisa, Italy; National Amyloidosis Centre, University College London, Royal Free Campus, London, UK; National Amyloidosis Centre, University College London, Royal Free Campus, London, UK; Health Sciences Interdisciplinary Center, Scuola Superiore Sant’Anna, piazza Martiri della Libertà 33, 56127 Pisa, Italy; Cardiology Division, Fondazione Toscana Gabriele Monasterio, via Moruzzi 1, 56124 Pisa, Italy; Health Sciences Interdisciplinary Center, Scuola Superiore Sant’Anna, piazza Martiri della Libertà 33, 56127 Pisa, Italy; Cardiology Division, Fondazione Toscana Gabriele Monasterio, via Moruzzi 1, 56124 Pisa, Italy

**Keywords:** Cardiac amyloidosis, Transthyretin, AL, Prevalence, Systematic review, Meta-analysis

## Abstract

**Background:**

Cardiac amyloidosis (CA) is an under-recognized cause of heart failure. Its prevalence in screening studies, and the extent to which selective confirmatory testing affects prevalence estimates, remain uncertain across clinical settings.

**Methods:**

We systematically searched PubMed/MEDLINE and EMBASE up to 10 August 2025. Two reviewers independently screened studies and extracted data. We grouped studies by clinical setting and combined prevalence estimates using random-effects meta-analysis. For each study, we calculated CA prevalence in (1) the whole enrolled cohort and (2) the subgroup who underwent confirmatory testing.

**Results:**

Eighty-three studies were included. Pooled CA prevalence (whole cohort; tested subgroup) was highest in left ventricular hypertrophy/hypertrophic cardiomyopathy [LVH/HCM] (15.1%; 35.4%), followed by heart failure (HF)—mainly HF with preserved or mildly reduced ejection fraction (HFpEF/HFmrEF)—(12.6%; 13.6%) and aortic stenosis (AS) (9.6%; 11.6%). Orthopaedic cohorts were lower overall (4.1%) but higher in tested subgroups (12.4%); ‘no specific red flags’ showed 1.8% vs. 7.8%; non-cardiac bone scintigraphy was 0.49% in both denominators. Across settings, transthyretin CA predominated over light-chain CA. Several studies approached systematic screening in the general elderly; however, they were few and still applied referral criteria to second-level examinations.

**Conclusion:**

This meta-analysis shows that CA is relatively frequent in HFpEF/HFmrEF, severe AS, and LVH/HCM. To obtain reliable population estimates, future studies should test either all eligible participants or a predefined random sample, rather than only those with suspected disease. In clinical practice, screening strategies should clearly define which higher-risk individuals are referred for second-level tests, balancing diagnostic yield with feasibility.

## Graphical Abstract

**Graphical Abstract:**
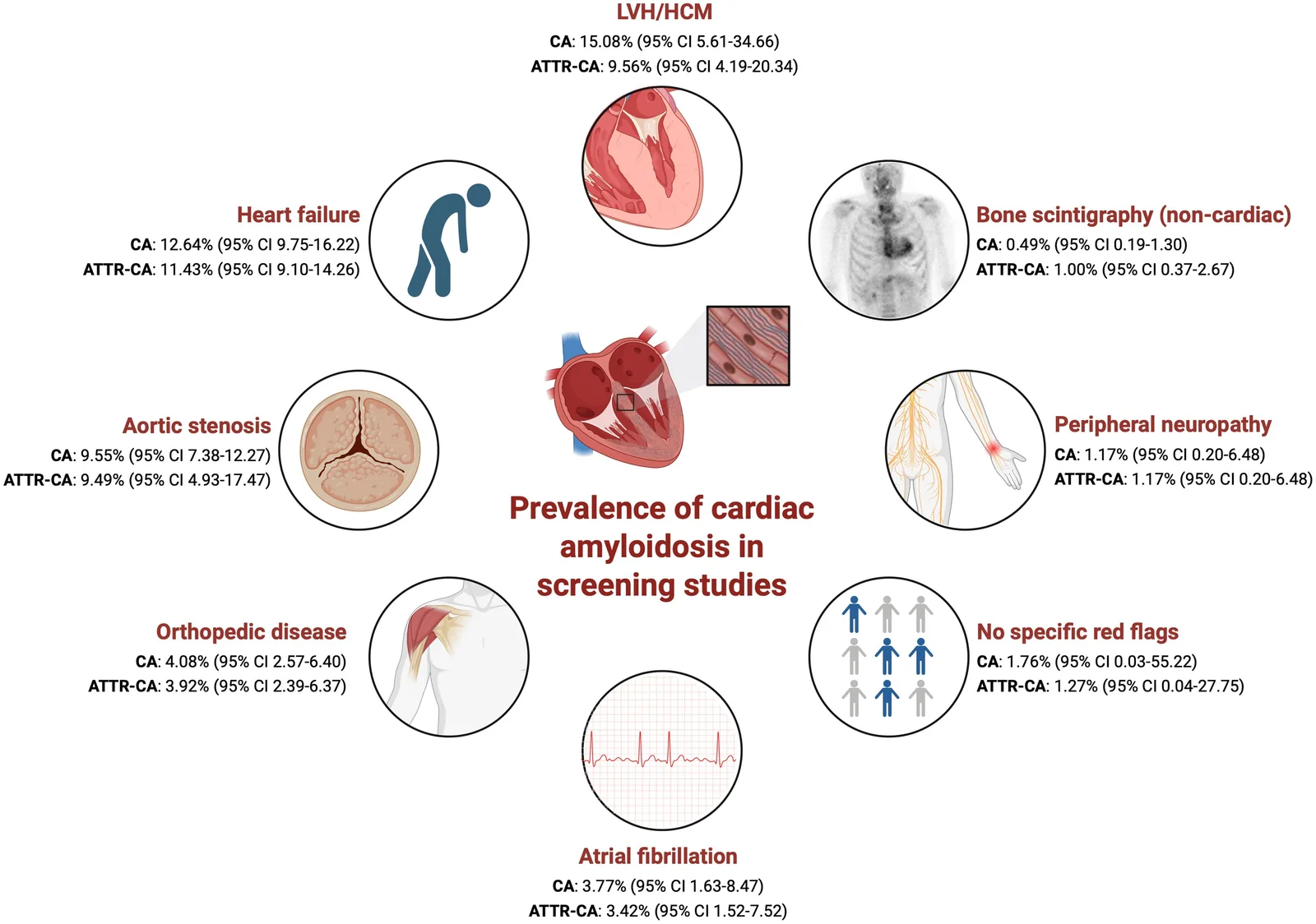
Screening yields of cardiac amyloidosis across clinical settings. Pooled prevalence of cardiac amyloidosis (CA) is shown for the whole enrolled cohort and for the subgroup undergoing confirmatory testing. ATTR-CA, transthyretin cardiac amyloidosis; AL-CA, light-chain cardiac amyloidosis; AS, aortic stenosis; HCM, hypertrophic cardiomyopathy; HF, heart failure; HFmrEF, heart failure with mildly reduced ejection fraction; HFpEF, heart failure with preserved ejection fraction; LVH, left ventricular hypertrophy.

## Introduction

Our understanding of the epidemiology of cardiac amyloidosis (CA) has largely come from administrative datasets and patient registries, which miss undetected disease.^1^ This evidence base has reinforced the notion of CA as a rare disease, defined as a condition affecting <5 per 10 000 in the European Union^2^ or fewer than 200 000 people in the USA,^3^ yet growing data suggest that case ascertainment in claims and registries is incomplete. Indeed, screening studies targeting populations with clinical ‘red flags’ or high-yield settings suggest a broader burden than historical coding-based estimates imply, particularly for transthyretin CA (ATTR-CA).^1^

Advances in non-invasive diagnosis and the availability of disease-modifying therapies have reshaped practice and given impulse to screening. Suspicion is often triggered by extra-cardiac manifestations (e.g. carpal tunnel syndrome [CTS] or peripheral neuropathy), discordant electrocardiographic (ECG)-echo findings (low voltages with increased left ventricular [LV] wall thickness), apical sparing on strain imaging, or Q waves without infarction, and by conditions with a strong epidemiological link to CA such as severe aortic stenosis (AS) and heart failure (HF) with preserved or mildly reduced ejection fraction (HFpEF/HFmrEF).^4^ Screening investigations across these settings have begun to redefine the epidemiology of CA, indicating a relatively high prevalence in HFpEF/HFmrEF, severe AS, or CTS cohorts, and in individuals undergoing bone scintigraphy for non-cardiac indications.^5^ However, prevalence estimates vary widely across studies, likely reflecting small sample sizes and substantial heterogeneity in enrollment enrichment and in the criteria used to refer participants for confirmatory testing of CA.

In 2022, two systematic reviews reappraised the available evidence about disease epidemiology.^5,6^ One focused specifically on screening studies and showed that targeted searches uncover substantial disease across multiple clinical contexts;^5^ another one included code-based cohorts, a strategy that risks underestimating true prevalence when active case finding is absent.^6^ Since those reports, new studies have explored additional settings, including unselected older adults,^7^ reinforcing that ATTR-CA in the general elderly population, while uncommon, may be more frequent than traditional ‘rare disease’ thresholds would suggest.

Building on this knowledge, we undertook a systematic review and meta-analysis to reappraise the prevalence of CA in screening studies. We synthesized data by clinical setting, amyloid subtype, and denominator (entire cohort vs. those undergoing confirmatory testing) to provide setting-specific pooled estimates and to clarify where targeted screening is most informative.

## Methods

### Literature search

We conducted a systematic search of PubMed/MEDLINE and EMBASE from inception to 10 August 2025. The core strategy combined prevalence/screening terms with amyloidosis terms in the title field: prevalence OR frequency OR epidemiology OR screening) AND (cardiac amyloidosis OR amyloidosis OR transthyretin OR ATTR OR light-chain OR AL); full database-specific strings (including filters and Boolean syntax) are reported in the Supplementary material. We imposed no date limits and restricted to English-language reports. To maximize retrieval, we hand-searched the reference lists of systematic reviews and meta-analyses,^5,6,8–12^ and a non-systematic review of studies on CA prevalence in HF.^13^ Records were de-duplicated prior to screening.

Eligibility criteria were prespecified. We included: (1) peer-reviewed full articles or research letters in English; (2) screening studies that actively searched for CA (defined as amyloid involvement of at least the LV) in clinical settings other than known systemic amyloidosis, carriers of pathogenic *TTR* variants, or first-degree relatives; and (3) studies reporting (or allowing back-calculation of) both a numerator (CA cases) and a denominator (either the entire enrolled cohort and/or the subgroup undergoing confirmatory testing). We excluded conference abstracts, preprints, or preclinical studies; studies limited to isolated valve or atrial amyloid without a CA work-up; cohorts in which ‘prevalence’ was derived only from administrative/claims codes without active case finding; studies of patients with suspected CA or established systemic CA; and reports from which CA prevalence could not be calculated. When multiple publications described overlapping cohorts, we retained the largest/most complete report and used companion papers only for supplementary details.

Two authors (A.A., V.C.) independently screened titles/abstracts and full texts against the criteria, with disagreements resolved by discussion and, if needed, adjudication by other authors (G.V., M.E.). Reasons for full-text exclusion were documented, and study selection is summarized in the PRISMA flow diagram. Following publication of a paper by the authors,^14^ this paper was added to the list, and an unstructured literature review was performed on 21 September 2025. No other relevant papers were found.

The review was designed and reported according to PRISMA 2020 criteria (checklist in the Supplementary material). The protocol was registered in the Open Science Framework (OSF) Registries prior to data extraction; no amendments were made after registration. The protocol is archived in OSF; the extracted dataset, data-collection form, and analysis code are available from the corresponding author upon reasonable request. The authors received no financial or non-financial support and have no conflicts of interest to disclose.

### Study classification

The studies were classified according to their population settings: (1) individuals from the general population and without specific red flags (possibly except for age), (2) individuals with one or more of the traditional red flags, regardless of the specific red flag(s), (3) any orthopaedic disease, (4) AS, (5) bone scintigraphy performed for non-cardiac reasons, (6) LV hypertrophy (LVH) or hypertrophic cardiomyopathy (HCM), (7) HF, (8) conduction disorders, (9) atrial fibrillation, (10) plasma cell disorders, (11) peripheral neuropathy, and (12) other specific settings. One study requiring two red flags^14^ was considered separately.

### Data extraction

Two authors (A.A. and V.C.) independently abstracted, for each study, the number enrolled, the number diagnosed with CA (overall and, when available, by subtype: ATTR-CA, AL-CA, and wild-type vs variant ATTR-CA), the sex distribution among CA cases, and the mean, median, or average age of CA patients. We recorded how each study selected participants and whether only a subset (for example, patients with specific ‘red flags’) was referred for confirmatory tests for CA (‘gatekeeping’). When only some participants were tested, we calculated two prevalence values: one using all enrolled participants as the denominator and another using only those who underwent confirmatory testing. If a study reported CA counts together with a prevalence percentage but not the denominator, we back-calculated the denominator from those quantities and rounded to the nearest integer; we flagged this as a potential source of minor rounding error. Risk of bias was assessed with the Joanna Briggs Institute (JBI) checklist for prevalence studies,^15^ in duplicate.

### Statistical analysis

We synthesized prevalence by clinical setting and, where available, by phenotype (overall CA, ATTR- or light-chain [AL]-CA) and by denominator (whole cohort vs further-tested subgroup). We summarized CA prevalence by clinical setting and, when available, by phenotype (overall CA, ATTR-CA, AL-CA) and by denominator (whole cohort vs further-tested subgroup). For settings with at least three studies, we combined study-specific proportions using random-effects meta-analysis on the logit scale. We calculated exact binomial 95% confidence intervals for individual studies and described between-study heterogeneity using Cochran’s *Q*, the *I*^2^ statistic, and the between-study variance (τ^2^). We applied Hartung–Knapp adjustments to obtain confidence intervals around pooled estimates. When studies used gatekeeping, we meta-analysed whole-cohort and tested-subgroup denominators separately. Certainty of evidence for each pooled prevalence estimate was assessed with an adapted GRADE approach, considering risk of bias, inconsistency, indirectness, imprecision, and publication bias. Forest plots display each study’s estimate with exact confidence intervals and the random-effects pooled estimate, with box sizes proportional to random-effects inverse-variance weights. When studies used gated verification, we meta-analysed the two denominators separately. The certainty of evidence for each pooled prevalence outcome was assessed using an adapted GRADE approach, starting at high certainty and rating down across five domains: risk of bias, inconsistency, indirectness, imprecision, and publication bias.

To explore sources of heterogeneity related to verification, we performed two complementary analyses. First, within each setting and denominator, we stratified studies by the presence of gatekeeping (defined as confirmatory testing applied only to a subset of enrolled participants) and produced separate pooled estimates for ‘gatekeeping present’ versus ‘no gatekeeping.’ Second, for studies reporting both denominators, we calculated the within-study inflation ratio (tested-subgroup prevalence divided by whole-cohort prevalence) and summarized its distribution across studies and settings. Influence was assessed by leave-one-out analysis within each setting and denominator: we re-pooled after omitting each study in turn and recorded the absolute change in the pooled prevalence (percentage points); we report the maximum and median leave-one-out shifts and provide study-level deltas.

To further explore between-study heterogeneity, we performed exploratory subgroup and meta-regression analyses in the two settings with the largest number of studies, namely HF and AS. Candidate study-level moderators were selected *a priori* based on clinical relevance and data availability: publication era, mean or median age, proportion of men, geographic region, diagnostic strategy, and use of gatekeeping for confirmatory testing. Publication era was categorized as pre-2015 versus 2015–2025. Geographic region was grouped as Europe, North America, Asia, or other/multiregional. Diagnostic strategy was categorized according to the main confirmatory approach used: bone scintigraphy-based, CMR/biopsy-based, or mixed. Univariable random-effects meta-regression was performed on logit-transformed prevalence estimates. Multivariable meta-regression was not performed because of the limited number of studies and collinearity between moderators, particularly publication era, diagnostic strategy, and gatekeeping. These analyses were considered exploratory and hypothesis-generating.

We also pooled three descriptive ratios by setting (ATTR:AL among CA diagnoses, the men-to-women ratio among CA cases, and the wild-type:variant ratio among ATTR-CA) by analysing log-ratios with a DerSimonian–Laird random-effects model (Haldane–Anscombe correction when a cell was zero) and back-transforming results to the ratio scale with 95% confidence intervals. Where only one study was available, we report the study’s ratio and the exact binomial confidence interval for the underlying proportion. Small-study effects were examined in settings with at least 10 studies by visually inspecting funnel plots of logit prevalence against its standard error and by testing asymmetry with Egger’s regression intercept and Begg–Mazumdar rank correlation; given high heterogeneity in several settings, these tests were interpreted cautiously.

All analyses were conducted in Python 3.11 (Jupyter). We used pandas for data handling, NumPy for numerical operations, SciPy for exact binomial intervals and random-effects routines (including our implementations of DerSimonian–Laird and Hartung–Knapp for pooled logits), Matplotlib for figures (forest, funnel, and influence plots), and XlsxWriter for tabular outputs. All tests were two-sided with α = 0.05.

## Results

### Screening studies identified

**Figure 1:**
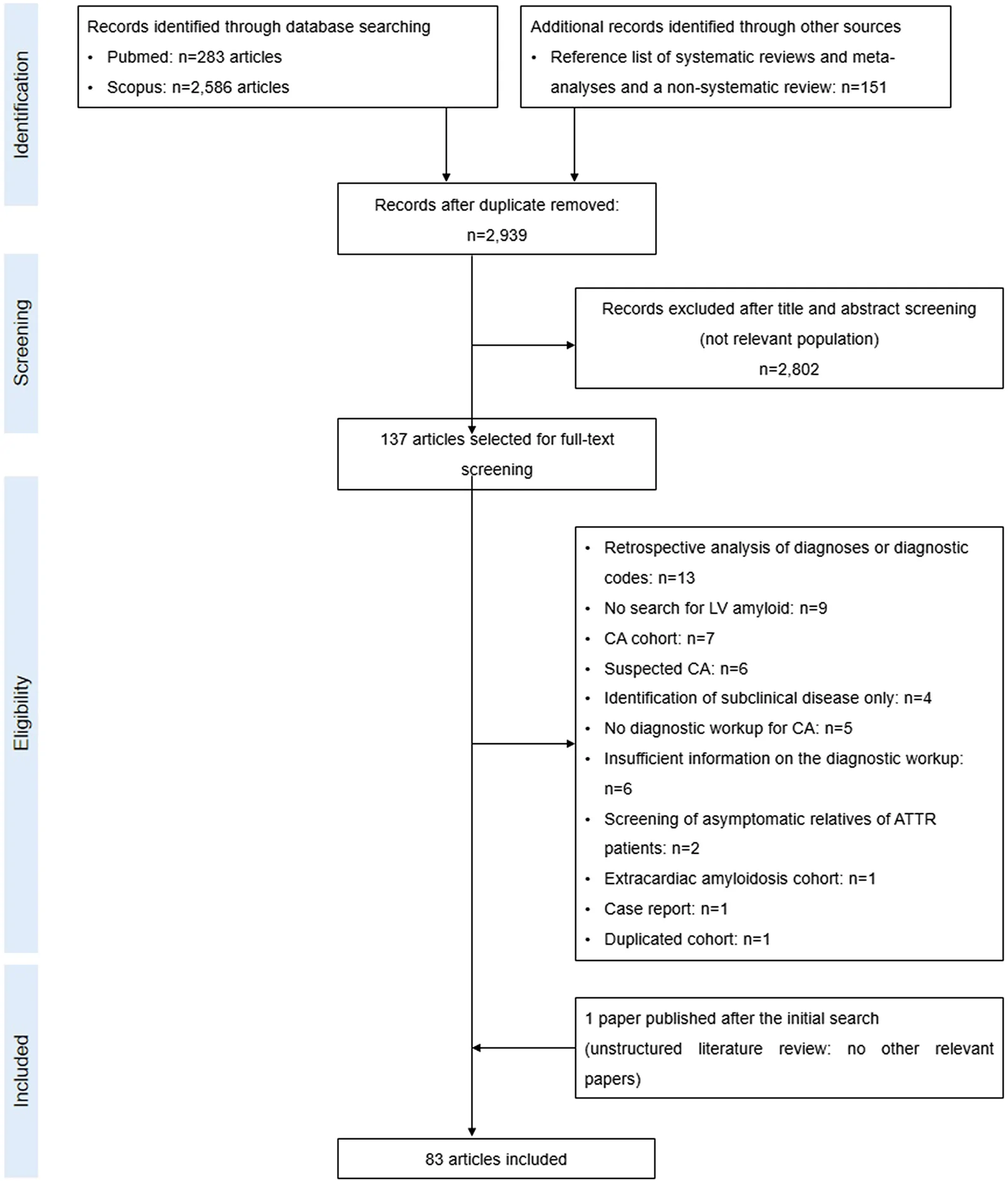
PRISMA 2020 flow diagram of study selection. Records were identified through database searches of PubMed/MEDLINE and EMBASE (search date: 10 Aug 2025), deduplicated, and screened in duplicate at title/abstract level. CA, cardiac amyloidosis

The process of study identification, screening, assessment of eligibility, and final selection is recapitulated in *Figure 1*. Eighty-three screening studies were ultimately selected.^7,13,16–94^ They were conducted in the following settings: HF (*n* = 20),^13,16,17,18,19,20,21,22,23,24,25,26,27,28,29,30,31,32,33,34^ AS (*n* = 16),^35,36,37,38,39,40,41,42,43,44,45,46,47,48,49,50^ orthopaedic disease (*n* = 14),^51,52,53,54–64^ bone scintigraphy for non-cardiac reasons (*n* = 12),^65–76^ LVH/HCM (*n* = 4),^77,78,79,80^ atrial fibrillation (*n* = 3),^81–83^ no specific red flags (*n* = 3),^7,84,85^ peripheral neuropathy (*n* = 3),^86–88^ one or more red flags (*n* = 2),^14,91,92^ two red flags (*n* = 1),^14^ conduction disorders (*n* = 2),^21,95^ plasma cell disorders (*n* = 2),^89,90^ or other specific setting (*n* = 2).^93,94^ Details about the study populations are provided in *Table 1*. Thirteen studies out of 20 on the HF setting investigated the HFpEF/HFmrEF phenotype.^13,16–18,20,22–24,27,31–34^ Studies on the AS setting were all conducted in individuals with severe disease,^35–38,40–50^ except for one including patients with moderate AS.^39^ Studies on orthopaedic disorders mainly enrolled patients with CTS,^51,52,54,55,57,59–62,64^ either evaluating only those patients^51,52,54,55,57,59,61,64^ or also those with other orthopaedic disorders,^60,62^ and often considering specific inclusion criteria (e.g. male individuals undergoing bilateral surgery).^55^ Several studies investigated cohorts of subjects undergoing bone scintigraphy for non-cardiac reasons (or mostly for non-cardiac reasons). Four studies investigated cohorts with LVH/HCM, but no HF.^77–80^ Two studies out of 3 in the AF setting investigated patients referred for ablation.^82,83^ The studies conducted in individuals without specific red flags involved the systematic assessment of subjects aged ≥75 years undergoing autopsy^84^ or those aged 65–90 years from the general population,^7^ or subjects ≥70 years visiting outpatient internal medicine clinics^85^; in the last two studies, the search for cardiac amyloid was conducted only in individuals with specific risk factors.^7,85^ In other two studies, only individuals with one or more red flags were enrolled;^91,92^ a third study enrolled subjects aged ≥65 years who had two red flags, namely CTS plus a history of HF and/or LVH.^14^ The cohorts of subjects with peripheral neuropathy were heterogeneous.^86–88^ One study enrolled patients with either conduction disorders or HF.^21^ Finally, we classified as studies performed in ‘other specific settings’ a study conducted in Black subjects from the Atherosclerosis Risk in Communities Study^93^ and a study on patients with cardiomyopathy referred to endomyocardial biopsy.^94^

**Table 1:** Studies selected

First author, year^ref^	Study setting	Study setting (details)	Single/multicentre	Subjects screened, n	Criteria for further testing for CA	Subjects undergoing further testing for cA
Mohammed, 2014^16^	Heart failure	Autopsy of patients with known HFpEF without clinically apparent amyloid	Single centre	109	None	109
González-López, 2015^17^	Heart failure	HF, EF ≥50%, LVH, age ≥60 years	Single centre	120	None	120
Bennani Smires, 2016^18^	Heart failure	HFpEF (EF >45%), >65 years, no CAD	Single centre	49	None	49
Arvanitis, 2017^19^	Heart failure	African Americans >60 years, diagnosis of HF, LV wall thickness ≥12 mm	Single centre	99	V122I genotype	5
Ton, 2017^20^	Heart failure	HFpEF referred for EMB	Single centre	259	None	259
López-Sainz, 2019^21^	Heart failure	Age ≥60 years, 1) indication to PM due to unexplained AV block, or 2) admission due to HF with EF <50% and LV end-diastolic wall thickness ≥12 mm	Single centre	28	None	28
Hahn, 2020^22^	Heart failure	HFpEF (EF ≥50%)	Single centre	108	None	108
AbouEzzedine, 2021^23^	Heart failure	HF, EF ≥40%, LVH, age ≥60 years	Multicentre (population-based cohort study)	286	None	286
Devesa, 2021^24^	Heart failure	HF, EF ≥50%, LV wall thickness <12 mm	Single centre	58	None	58
Goland, 2021^25^	Heart failure	Unexplained LV dysfunction (EF <50%)	Single centre	75	None	75
Lindmark, 2021^26^	Heart failure	HF, LVH	Single centre	86	None	86
Jaramillo-Hidalgo, 2023^27^	Heart failure	Age ≥75 years, clinical history of HFpEF, atrial dilation ≥34 ml/m^2^ and LV wall thickening >13 mm	Single centre	50	None	50
Madhani, 2023^28^	Heart failure	HF, Black and Hispanic, ≥60 years	Multicentre	278	None	278
Ruiz-Hueso, 2023^29^	Heart failure	age ≥65 years, HF, interventricular septum or posterior wall thickness >12 mm	Multicentre	453	None	453
Spaccavento, 2024^30^	Heart failure	Age ≥60 years, admitted for acute HF	Single centre	103	None	103
Tubben, 2024^31^	Heart failure	NYHA II–III), EF >40%, septal or posterior wall thickness ≥11 mm, and/or mean septal and lateral e’ <9 cm/s or E/e’ ≥13 and/or a left atrial volume index ≥34 ml/m^2^	Multicentre	104	None	104
Yun, 2024^32^	Heart failure	HF, EF >40%, LVH, age ≥60 years	Multicentre	315	None	315
Achten, 2025^33^	Heart failure	HFpEF (EF ≥50%)	Single centre	202	None	202
Garcia-Pavia, 2025^13^	Heart failure	HF, EF ≥50%, LVH, ≥50 years	Multicentre	387	None	387
Healy, 2025^34^	Heart failure	HF, EF ≥50%, LVH, ≥60 years, no prior evaluation for CA or known monoclonal gammopathy	Single centre	86	None	86
Nietlispach, 2012^35^	Aortic stenosis	Patients with prior TAVI who died and underwent a post-mortem examination	Single centre	20	None	20
Longhi, 2016^36^	Aortic stenosis	Aortic stenosis referred for AVR	Single centre	43	≥1 echo ‘red flags’: increased thickness of atrioventricular valves, interatrial septum or right ventricular free wall, pericardial effusion, and myocardial granular sparkling	5
Treibel, 2016^37^	Aortic stenosis	Severe aortic stenosis referred for AVR	Single centre	146	None	146
Castano, 2017^38^	Aortic stenosis	Severe aortic stenosis undergoing TAVR	Single centre	151	None	151
Cavalcante, 2017^39^	Aortic stenosis	Moderate or severe aortic stenosis undergoing CMR as clinically indicated	Single centre	113	None	113
Nitsche, 2020^40^	Aortic stenosis	Severe aortic stenosis scheduled for TAVR	Single centre	191	None	191
Oda 2020^41^	Aortic stenosis	Severe aortic stenosis undergoing CT before TAVR	Single centre	192	Clinical and echo red flags	25
Scully, 2020^42^	Aortic stenosis	Severe aortic stenosis referred for TAVR, age ≥75 years	Multicentre	200	None	200
Nitsche, 2021^43^	Aortic stenosis	Severe aortic stenosis referred for TAVR	Multicentre	407	None	407
Rosenblum, 2021^44^	Aortic stenosis	Severe aortic stenosis undergoing TAVR	Multicentre	204	None	204
Shimoni, 2021^45^	Aortic stenosis	Recent TAVR	Single centre	88	None	88
Singal, 2021^46^	Aortic stenosis	Symptomatic severe aortic stenosis patients undergoing SAVR, age ≥65 years	Single centre	32	None	32
Costa, 2024^47^	Aortic stenosis	TAVR	Single centre	100	None	100
Jakstaite, 2024^48^	Aortic stenosis	Severe aortic stenosis, ≥65 years, ≥1 major (CTS, ruptured biceps tendon, spinal stenosis, NT-proBNP ≥1000 ng/L, cardiac troponin >99th percentile) or ≥2 minor criteria (diastolic dysfunction ≥2 grade/lateral e’ <10 cm/s, atrial fibrillation, atrioventricular conduction disease/pacemaker)	Single centre	85	None	85
SadrAldin, 2024^49^	Aortic stenosis	Severe aortic stenosis undergoing SAVR or TAVR	Single centre	27	None	27
McShane, 2025^50^	Aortic stenosis	Severe aortic stenosis patients undergoing TAVR	Single centre	161	ECV ≥31%	30
Sperry, 2018^51^	Orthopaedic disease	CTS surgery, ≥50 years (M), ≥60 years (W)	Single centre	98	Amyloid deposition in the surgical specimen	10
Zegri-Reiriz, 2019^52^	Orthopaedic disease	CTS surgery, ≥60 years	Single centre	233	LVH	101
Sperry, 2021^53^	Orthopaedic disease	Surgery for idiopathic trigger finger, age ≥50 years	Single centre	100	Amyloid deposition in the surgical specimen	2
Sugiura, 2021^54^	Orthopaedic disease	CTS surgery	Single centre	79	Amyloid deposition in the surgical specimen	27
Vianello, 2021^55^	Orthopaedic disease	Males, bilateral CTS surgery	Single centre	53	LVH	6
Baylor, 2022^56^	Orthopaedic disease	Surgery for distal biceps tendon rupture	Single centre	30	Amyloid deposition in the surgical specimen	1
Westin, 2022^57^	Orthopaedic disease	Surgery for bilateral CTS 5–15 years before	Multicentre (recruitment from nationwide registries)	250	None	250
Debonnaire, 2023^58^	Orthopaedic disease	Symptomatic SS surgery	Single centre	82	None	82
Takashio, 2023^59^	Orthopaedic disease	CTS surgery	Single centre	120	(1) IVSd ≥14 mm or (2) 12 mm ≤IVSd <14 mm with above-normal limits in hs-TnT	120
Andrei, 2024^60^	Orthopaedic disease	CTS, atraumatic biceps tendon rupture, lumbar spinal stenosis; age ≥60 years	Single centre	57	None	57
Gannon, 2024^61^	Orthopaedic disease	CTS surgery +‘appropriateness screening criteria’ for CA	Single centre	62	Amyloid deposition in the surgical specimen	14
Yamada, 2024^62^	Orthopaedic disease	Arthroscopic shoulder surgery or CTS surgery	Single centre	41	Amyloid deposition in the surgical specimen	3
Eldhagen, 2025^63^	Orthopaedic disease	Lumbar spinal stenosis surgery 6 years before	Single centre	19	None	19
Pimenta, 2025^64^	Orthopaedic disease	Symptomatic CTS requiring hand surgery, age ≥60 years	Single centre	22	None	22
al-Nahhas, 1995^65^	Bone scintigraphy for non-cardiac reasons	Patients with prosthatic cancer	Single centre	965	None	965
Longhi, 2014^66^	Bone scintigraphy for non-cardiac reasons	Bone scan for non-cardiac indications	Single centre	12 400	None	12 400
Mohamed-Salem, 2018^67^	Bone scintigraphy for non-cardiac reasons	Bone scan for non-cardiac indications, ≥75 years	Single centre	1114	None	1114
Kim, 2019^68^	Bone scintigraphy for non-cardiac reasons	Bone scan for non-cardiac indications, >30 years	Single centre	9581	None	9581
Bianco, 2021^69^	Bone scintigraphy for non-cardiac reasons	Bone scintigraphy for any reason	Single centre	4228	None	4228
Cuscaden, 2021^70^	Bone scintigraphy for non-cardiac reasons	Bone scan for non-cardiac indications	Single centre	3472	None	3472
Halme, 2022^71^	Bone scintigraphy for non-cardiac reasons	Bone scintigraphy for any reason (1.9% for suspected CA)	Multicentre	1334	None	1334
Nitsche, 2022^72^	Bone scintigraphy for non-cardiac reasons	Bone scintigraphy for any reason (90.6% for non-cardiac indications)	Single centre	10 443	None	10 443
Salvalaggio, 2022^73^	Bone scintigraphy for non-cardiac reasons	Bone scintigraphy for any reason	Single centre	9616	None	9616
Suomalainen, 2022^74^	Bone scintigraphy for non-cardiac reasons	Bone scintigraphy for any reason, >70 years	Multicentre	2000	None	2000
Navarro-Saez, 2023^75^	Bone scintigraphy for non-cardiac reasons	Bone scintigraphy for any reason, no known CA	Single centre	3554	None	3554
Son, 2024^76^	Bone scintigraphy for non-cardiac reasons	Bone scan for non-cardiac indications	Single centre	32 245	None	32 245
Cariou, 2017^77^	LVH/HCM	Subjects undergoing diagnostic workup for LVH	Single centre	114	None	114
Maurizi, 2020^78^	LVH/HCM	Tentative HCM diagnosis, age ≥40 years	Multicentre	343	At least one red flag for CA	26
Nakao, 2021^79^	LVH/HCM	Subjects undergoing diagnostic workup for LVH	Multicentre	295	None	295
Merlo, 2022^80^	LVH/HCM	Subjects ≥55 years referred to echo for any reasons (except those with known or suspected CA) with LVH, no dilation, EF ≥50%	Multicentre	1169	Echocardiographic red flags	217
Remior-Pérez, 2025^81^	Atrial fibrillation	Nonvalvular atrial fibrillation from <1 year, ≥1 red flag for CA, age ≥65 years	Multicentre	121	None	121
Yamasaki, 2024^82^	Atrial fibrillation	Atrial fibrillation referred for ablation	Single centre	342	Red-flag signs defined by echocardiographic or electrocardiographic findings, medical history, symptoms, and blood biochemical findings	69
Shinzato, 2025^83^	Atrial fibrillation	Atrial fibrillation referred for ablation	Single centre	385	None	385
Porcari, 2021^84^	No specific red flag	Subjects ≥75 years undergoing autopsy	Single centre	56	None	56
Aimo, 2024^7^	No specific red flag	Subjects aged 65–90 years from the general population	Single centre	1000	Clinical criteria: history of CTS, spontaneous tendon rupture, lumbar spine stenosis, any known plasma cell dyscrasia and/or symptoms compatible with peripheral or autonomic neuropathy; ECG criteria: low QRS voltage or disproportionately low compared with LV mass and/or pseudo-Q waves; echo criteria: LV thickness ≥ 12 mm and/or diastolic dysfunction grade 2 and 3 and/or a history of severe aortic stenosis; laboratory criterion: hs-TnT ≥14 ng/L	216
Arima, 2025^85^	No specific red flag	Subjects ≥70 years visiting outpatient internal medicine clinics	Multicentre	1141	hs-cTnT ≥30 ng/L	55
Di Stefano, 2024^86^	Peripheral neuropathy	Sensory–motor idiopathic polyneuropathy and two or more ‘red flags’	Single centre	145	None	145
Doneddu, 2025^87^	Peripheral neuropathy	Chronic inflammatory demyelinating polyradiculoneuropathy	Multicentre	124	≥1 red flag for *TTR* gene variants	81
Samuelsson, 2019^88^	Peripheral neuropathy	Idiopathic small-fiber neuropathy or mixed neuropathy	Multicentre	155	None	155
de Frutos, 2024^91^	One or more red flags	≥1 red flag for ATTRv: unexplained HF, LV hypertrophy, PM implantation, peripheral neuropathy, CTS, lumbar spinal stenosis	Single centre	256	None	256
Di Stefano, 2025^92^	One or more red flags	≥2 major red flags for ATTRv (age >65 years, progressive sensory or sensorimotor neuropathy not responsive to steroids or immunomodulant therapies, recent and unexplained weight loss + GI disease, diagnosis of CA, bilateral or relapsing CTS, unexplained autonomic dysfunction) or one major flag and two minor flags (family history of neuropathy, ambulation disorders or cardiopathy, SCD, a bedridden, wheelchaired patient without specific diagnosis, infections, juvenile cardiac disease, ocular disorders, lumbar spine stenosis, biceps tendon rupture)	Single centre	29	None	29
Aimo, 2025^14^	Two red flags	Subjects aged ≥65 years with CTS and HF ± CTS	Multicentre	1215	None	1215
López-Sainz, 2019^21^	Conduction disorders	Age ≥60 years, (1) indication to PM due to unexplained AV block, or (2) admission due to HF with EF <50% and LV end-diastolic wall thickness ≥12 mm	Single centre	111	None	111
Cannie, 2024^95^	Conduction disorders	Age 70–85 years, PM implantation for idiopathic high-degree AV block	Single centre	39	None	39
Mangiacavalli, 2025^89^	Plasma cell disorders	Intermediate/high-risk MGUS	Single centre	1375	None	1375
Zhou, 2025^90^	Plasma cell disorders	Lambda SMM or MGUS, dFLC >23 mg/L, age >60 years	Multicentre	30	None	30
Quarta, 2015^93^	Other specific setting	Black subjects (ARIC cohort), *TTR* gene analysis, available echo	Multicentre	1240	None	1240
Tebbe, 2016^94^	Other specific setting	Unspecified CMP referred for EMB	Single centre	57	None	57

ATTR, amyloid transthyretin; AV, atrioventricular; CA, cardiac amyloidosis; CAD, coronary artery disease; CMP, cardiomyopathy; CMR, cardiac magnetic resonance; CTS, carpal tunnel syndrome; dFLC, difference between involved and non-involved free light chains; ECG, electrocardiogram; ECV, extracellular volume; EF, ejection fraction; EMB, endomyocardial biopsy; GI, gastrointestinal; HCM, hypertrophic cardiomyopathy; HF, heart failure; HFpEF, heart failure with preserved ejection fraction; hs-TnT, high-sensitivity troponin T; IVSd, interventricular septal thickness in diastole; LV, left ventricular; LVH, left ventricular hypertrophy; M, men; MGUS, monoclonal gammopathy of unknown significance; NT-proBNP, N-terminal pro-B-type natriuretic peptide; NYHA, New York Heart Association; PM, pacemaker; SAVR, aurgical aortic valve replacement; SCD, sudden cardiac death; SMM, smoldering myeloma; TAVR, transcatheter aortic valve replacement; v, variant; W, women.

### Prevalence of cardiac amyloidosis

The estimated prevalence values from the 83 studies are reported in Supplementary Table S1. For the studies where only a subgroup of patients was selected for further testing for CA, prevalence values in the whole cohort and in subjects undergoing further testing are reported. Pooled prevalence values for the settings with at least 3 studies are reported in *Table 2* and are visually represented in *Figure 2*. Forest plots for the specific settings are provided in Supplementary Figures S1–16.

**Figure 2:**
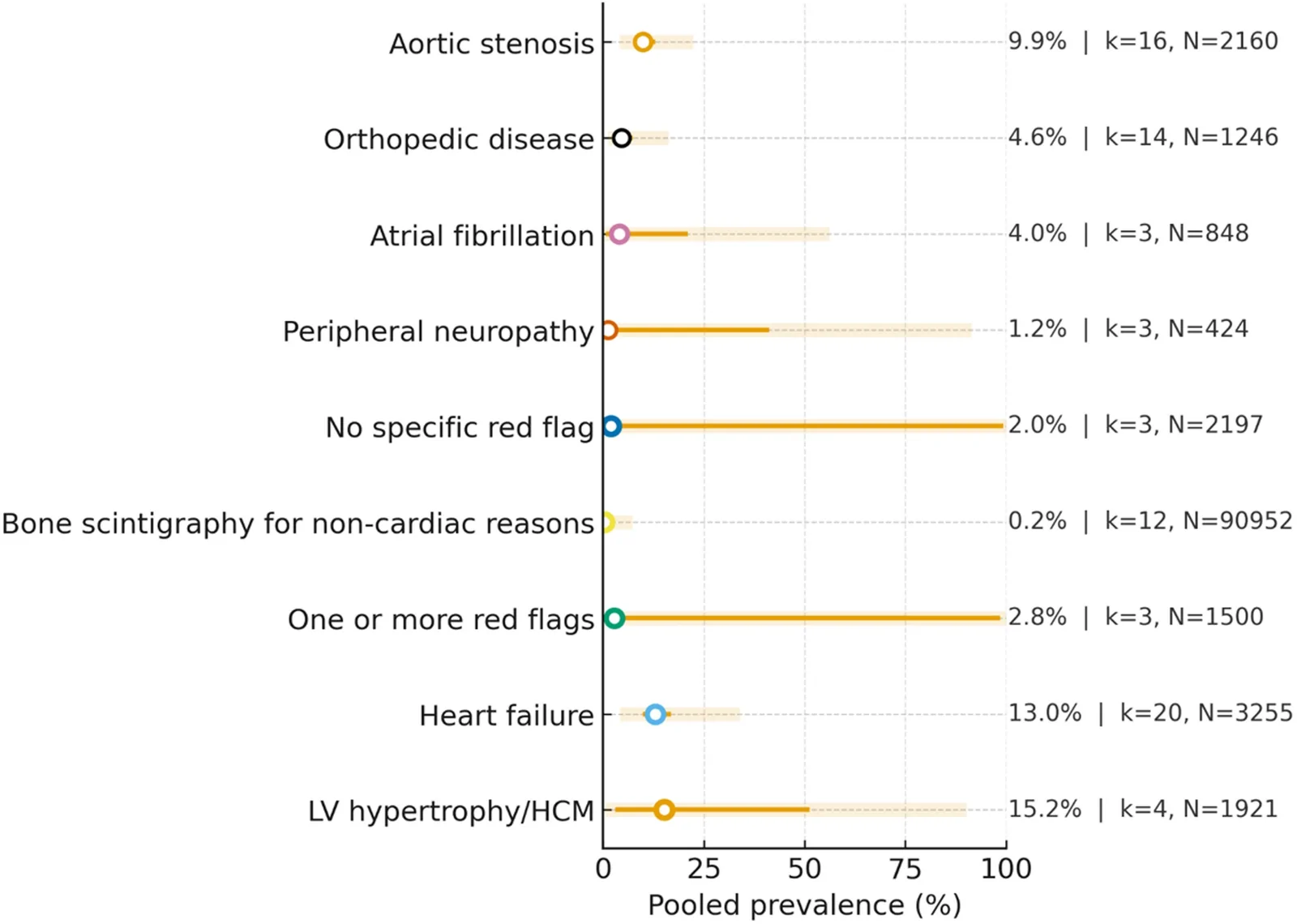
Prevalence of cardiac amyloidosis (CA) in whole cohorts. Settings with ≥3 studies are considered. Points are pooled estimates from random-effects meta-analysis on the logit scale. Thin horizontal lines show 95% confidence intervals (CIs); thick faint bars show 95% prediction intervals (PIs). Right-side labels report the pooled prevalence and k, N (number of studies; total participants). Point outline thickness encodes heterogeneity (I^2^). AS, aortic stenosis; HF, heart failure; HFpEF/HFmrEF, HF with preserved/mildly reduced ejection fraction; LVH/HCM, left ventricular hypertrophy/hypertrophic cardiomyopathy

**Table 2:** Prevalence of cardiac amyloidosis (CA) in the different settings

Setting	Whole cohorts			Further testing for CA		
	CA (%)	ATTR-CA (%)	AL-CA (%)	CA (%)	ATTR-CA (%)	AL-CA (%)
Heart failure	12.64 (9.75–16.22)	11.43 (9.10–14.26)	0.83 (0.33–2.09)	13.58 (10.58–17.28)	12.17 (9.77–15.07)	0.94 (0.38–2.34)
Aortic stenosis	9.55 (7.38–12.27)	9.49 (4.93–17.47)	0.46 (0.23–0.92)	11.62 (9.10–14.72)	13.75 (7.35–24.28)	0.63 (0.31–1.27)
Orthopaedic disease	4.08 (2.57–6.40)	3.92 (2.39–6.37)	0.77 (0.39–1.55)	12.44 (6.50–22.50)	11.62 (5.92–21.55)	2.51 (1.20–5–17)
Bone scintigraphy for non-cardiac reasons	0.49 (0.19–1.30)	1.00 (0.37–2.67)	0.02 (0.01–0.07)	0.49 (0.19–1.30)	1.00 (0.37–2.67)	0.02 (0.01–0.07)
LVH/HCM	15.08 (5.61–34.66)	9.56 (4–19–20.34)	3.39 (0.84–12.67)	35.37 (20.26–54.12)	25.46 (11.49–47.35)	9.00 (5.19–15.15)
Atrial fibrillation	3.77 (1.63–8.47)	3.42 (1.52–7.52)	0.40 (0.13–1.25)	6.04 (3.13–11.36)	5.55 (2.68–11.13)	0.53 (0.17–1.62)
No specific red flag	1.76 (0.03–55.22)	1.27 (0.04–27.75)	0.45 (0–41.46)	7.76 (0.58–54.91)	5.70 (0.92–28.23)	2.17 (0.08–38.06)
Peripheral neuropathy	1.17 (0.20–6.48)	1.17 (0.20–6.48)	0.35 (0.07–1.74)	1.41 (0.29–6.55)	1.41 (0.29–6.55)	0.41 (0.08–2.00)

Prevalence and 95% confidence interval values are reported for the settings with at least three studies. Prevalence values were calculated separately out of the whole cohorts and out of the subjects undergoing further testing for cardiac amyloidosis (CA; see *Table 2*). AL, amyloid light-chain; ATTR, amyloid transthyretin; HCM, hypertrophic cardiomyopathy; LVH, left ventricular hypertrophy.

The highest prevalence of CA was found in patients with LVH/HCM (15.08% in the whole cohorts and 35.37% in the subgroups undergoing further testing), followed by HF (12.64% and 13.58%, respectively) and AS (9.55% and 11.62%, respectively) (*Table 2*). We also considered separately the studies on the HFpEF/HFmrEF setting, which yielded a pooled prevalence of CA of 13.40% (8.49–20.51%) in both the whole cohorts and the subgroups undergoing further testing. In studies investigating the CTS setting, the prevalence of CA was 3.95% (2.19–7.02%) in the whole cohorts and 13.47% (5.04–31.34%) in the subgroups undergoing further testing.

In patients with orthopaedic disorders, the prevalence values were 4.08% and 12.44%, respectively. As for unselected subjects, the prevalence values were 1.76% and 7.76%, respectively, in patients without red flags, and 0.49% and 0.49%, respectively, in patients undergoing bone scintigraphy for non-cardiac reasons (*Table 2*).

In all settings, the total prevalence of CA was driven by the prevalence of ATTR-CA, which was much more common of AL-CA. The ATTR:AL ratio was highest in the bone scintigraphy setting (43.65), followed by AS (20.59) and HF or LVH/HCM (both 12.30) (*Table 3*). The highest male-to-women ratio was found in the bone scintigraphy setting (4.05), followed by AS (2.77), LVH/HCM (2.61), and HF (2.10). Interestingly, the ratio was just 1.09 in the studies where subjects were enrolled regardless of red flags (Supplementary Table S2). The ratio between wild-type and variant disease was also considered and was highest in the AS setting (26.35) and lowest in the LVH/HCM setting (2.73); in the studies enrolling without red flags, it was 6.78 (Supplementary Table S3).

**Table 3:** Ratio between diagnoses of amyloid transthyretin (ATTR) and amyloid light-chain (AL) cardiac amyloidosis (CA)

Setting	ATTR:AL ratio (95% CI)
Heart failure	12.30 (5.07–29.83)
Aortic stenosis	20.59 (9.98–42.48)
Orthopaedic disease	4.29 (1.91–9.66)
Bone scintigraphy for non-cardiac reasons	43.65 (13.26–143.70)
LVH/HCM	12.30 (5.10–29.75)
Atrial fibrillation	8.01 (2.39–26.80)
No specific red flag	1.89 (0.49–7.27)
Peripheral neuropathy	3.15 (0.43–23.31)

HCM, hypertrophic cardiomyopathy; LVH, left ventricular hypertrophy.

### Subgroup analyses, study heterogeneity, risk of bias, publication bias

See Supplementary material.

## Discussion

This systematic review and meta-analysis show that CA prevalence varies widely by clinical setting and by denominator, yet is consistently non-negligible in several high-yield contexts—supporting with reasonable certainty that CA is not rare. Across whole cohorts, pooled prevalence was highest in LVH/HCM, HF (predominantly HFpEF/HFmrEF), and AS and was generally similar or higher when restricted to participants who underwent confirmatory testing. Within the HF spectrum, HFpEF/HFmrEF yielded ≈13% whether calculated in entire cohorts or among further-tested subgroups. In orthopaedic cohorts (predominantly CTS), prevalence was modest in whole cohorts but rose substantially among patients selected for confirmation, whereas individuals referred for bone scintigraphy for non-cardiac reasons had very low prevalence. Across settings, ATTR-CA predominated over AL-CA; however, the relative contribution of AL-CA differed substantially across clinical contexts, as reflected by the much lower ATTR:AL ratio in individuals without specific red flags compared with AS, and by the relatively greater AL-CA contribution in orthopaedic cohorts. Wild-type disease was more common than variant ATTR in AS and, to a lesser extent, in HF; and men were over-represented except in unselected populations, where the sex ratio approached unity (*Graphical Abstract*).

These observations align with disease biology and referral pathways. HFpEF/HFmrEF, severe AS, and LVH/HCM enrich for older patients and for myocardial phenotypes in which extracellular deposition and increased wall thickness heighten suspicion for CA. The particularly high ATTR:AL ratio in AS likely reflects the selective enrichment of this setting for older, often male patients with degenerative valve disease, a phenotype closely linked to wild-type ATTR-CA. Conversely, the lower ATTR:AL ratio in populations without specific red flags suggests that less selected elderly cohorts capture a more biologically diverse CA landscape, including a higher relative burden of AL-CA. Orthopedic ‘red flags’ such as CTS detect extracardiac deposits; when combined with cardiac hypertrophy or other cardiac red flags, diagnostic yield increases. The relatively higher contribution of AL-CA in orthopaedic cohorts is also clinically relevant because tendon or ligament amyloid deposition should not be assumed to represent ATTR disease only. By contrast, non-cardiac scintigraphy cohorts dilute pre-test probability.

Pooled prevalence estimates should be interpreted in light of substantial between-study heterogeneity. The highest pooled CA prevalence was observed in cohorts with LVH/HCM, followed by HF and AS; however, heterogeneity was extreme in LVH/HCM and substantial in HF and AS. Therefore, these estimates should be viewed as setting-level indicators of diagnostic yield rather than precise prevalence values applicable to all patients within each category. Heterogeneity was pervasive and is central to the interpretation of this meta-analysis. It likely reflects several interacting factors rather than random variation alone. First, age and sex composition differed across settings and across individual studies, and both are strongly related to ATTR-CA prevalence. Second, geographic origin may influence subtype distribution, particularly because the prevalence of TTR variants differs across populations. Third, publication era is relevant because studies conducted before widespread adoption of contemporary non-invasive diagnostic algorithms may have had lower sensitivity for CA detection. Fourth, diagnostic pathways differed substantially: some studies used systematic bone scintigraphy and monoclonal protein testing, whereas others relied on CMR, biopsy, autopsy, or mixed approaches. Finally, the criteria used to trigger confirmatory testing varied widely, ranging from universal testing to highly selective gatekeeping based on imaging, biomarkers, or extracardiac red flags. These differences mean that pooled estimates should not be interpreted as fixed prevalence values, but rather as approximate measures of diagnostic yield within broad clinical contexts. In the HF and AS settings, where the number of studies was largest, heterogeneity remained substantial even after stratification by denominator. In HF, variability probably reflects differences in HF phenotype, EF thresholds, LV wall-thickness criteria, age, race/ethnicity, and the degree to which cohorts represented HFpEF/HFmrEF rather than broader HF populations. In AS, heterogeneity likely reflects differences in AS severity, TAVR versus SAVR populations, age and sex composition, geographic setting, and whether confirmatory testing was performed systematically or only after red-flag screening. These observations support the use of context-specific screening algorithms rather than a single pooled prevalence estimate to guide clinical decision-making.

The LVH/HCM category requires careful interpretation. Although these cohorts yielded the highest pooled prevalence, only four studies contributed to this estimate, and they did not represent a single clinical population. Three studies enrolled patients with LVH or unexplained wall thickening, whereas one enrolled patients with a tentative HCM diagnosis. These populations differ clinically: unexplained LVH is often part of the diagnostic work-up for infiltrative cardiomyopathy, whereas established or genetically mediated HCM has a different pre-test probability for CA. Pooling these groups may therefore overstate the generalizability of the estimate and risks presenting a mixed diagnostic category as a single disease entity. We therefore consider the LVH/HCM pooled estimate exploratory and best interpreted as evidence that CA should be actively considered in patients referred for increased LV wall thickness, particularly when hypertrophy is unexplained or accompanied by amyloid red flags.

The ‘no specific red flags’ category deserves particular caution because it is the closest available approximation to population-level screening but also the least internally homogeneous setting. The three available studies address different questions rather than estimating the same parameter. The autopsy study provides evidence on concealed CA in very elderly deceased individuals and may capture disease missed during life, but it cannot be directly translated into prevalence among living community-dwelling older adults. The general population screening study provides the most relevant framework for public health purposes because participants were recruited from the community; however, only those meeting predefined clinical, ECG, echocardiographic, or biomarker criteria proceeded to second-level testing, so the whole-cohort estimate remains affected by partial verification. The outpatient internal medicine clinic study enrolled older healthcare users and used a biomarker-based gatekeeping strategy, representing an intermediate population between community screening and clinically enriched cohorts. These design differences explain the extremely wide confidence intervals and make the pooled estimate for this category largely exploratory rather than a reliable population prevalence estimate.

Only a few studies attempted systematic screening in unselected older adults, and even these referred only some participants for second-line tests. This finding underscores the major influence of selective confirmatory testing on prevalence estimates. We therefore make two practical recommendations. First, to obtain reliable population estimates, future all-comer studies should either test all eligible participants or test a predefined random sample, rather than only those with red flags. Ideally, such studies should be prospective, multicenter, and population-based; enroll a sufficiently large age-stratified sample of community-dwelling older adults; apply the same first-line assessment to all participants; and ensure that either all participants or a prespecified random subset undergoes complete confirmatory testing, including monoclonal protein assessment and cardiac imaging/bone scintigraphy according to standardized criteria. They should also report participation rates, reasons for non-participation, amyloid subtype, and prevalence estimates using clearly defined denominators. Without this design, it will remain difficult to distinguish true population prevalence from the effects of referral, survivorship, healthcare use, and gatekeeping. Second, in routine care, it is reasonable to reserve second-line tests (bone scintigraphy plus monoclonal protein testing) for patients at higher risk. Importantly, the amyloid-subtype distribution across settings argues against ATTR-only screening pathways. In broader elderly populations and in orthopaedic red-flag cohorts, first-line testing should systematically include monoclonal protein assessment—serum free light chains plus serum and urine immunofixation—alongside imaging-based evaluation, to avoid missing AL-CA. The chosen red-flag criteria should be clearly defined and tailored to local resources, aiming for a transparent balance between sensitivity and feasibility.

The sex distribution across settings also deserves specific attention. Although men were over-represented among CA cases in most clinically enriched cohorts, the male-to-female ratio approached unity in studies without specific red flags, whereas it was highest in cohorts undergoing bone scintigraphy for non-cardiac indications. This contrast may reflect, at least in part, true biological differences in disease expression; however, it also raises the possibility that current red-flag criteria and referral pathways preferentially detect CA in men. Women with CA may have less marked absolute LV wall thickening, smaller ventricular dimensions, different biomarker profiles, or symptoms more readily attributed to HFpEF, aging, or non-amyloid comorbidity. In addition, fixed wall-thickness thresholds may be less sensitive in women because they do not account for sex-related differences in cardiac size. These findings therefore support the need for sex-aware screening strategies, including careful interpretation of LV wall thickness relative to body size and sex, attention to extracardiac red flags in women, and lower reliance on ‘classic’ male-predominant phenotypes. Future studies should report sex-stratified prevalence, diagnostic pathways, and test performance to determine whether modified screening algorithms can reduce underdiagnosis of CA in women.

These findings have direct clinical and organizational implications. Screening should be prioritized where pooled prevalence and actionability are highest (HFpEF/HFmrEF, severe AS, LVH/HCM) using standardized first-line algorithms; CTS pathways should incorporate prompts to evaluate for CA in older adults—especially with bilateral CTS or coexisting LVH. In AS, the high ATTR:AL ratio supports a strong role for bone scintigraphy in the diagnostic pathway, but monoclonal protein testing remains essential to exclude AL-CA and to correctly classify amyloid subtype. In orthopaedic and less selected elderly cohorts, the relatively greater AL-CA contribution reinforces the need for simultaneous ATTR and AL assessment from the outset. The predominance of contemporary studies also means that the pooled estimates are most applicable to current diagnostic practice, whereas older studies should be interpreted in light of less sensitive and less standardized diagnostic pathways. This is particularly relevant because currently available disease-modifying therapies for CA, especially ATTR-CA, mainly aim to slow or prevent disease progression rather than reliably reverse established myocardial damage. Therefore, adequately designed screening campaigns may be crucial to identify patients at an earlier, more treatable stage, before advanced cardiac involvement limits the potential benefit of therapy. Service planning should anticipate demand for confirmatory diagnostics and for genetic counseling in variant ATTR. As recognition of CA increases, access to disease-modifying therapies will require transparent criteria based on disease stage and expected benefit, together with shared decision-making about absolute gains and opportunity costs.

Limitations include substantial-to-extreme between-study heterogeneity despite stratification, frequent partial verification, occasional reconstruction of denominators from reported percentages, variability in diagnostic era and subtype ascertainment, and possible small-study effects. Although exploratory subgroup and meta-regression analyses were performed in HF and AS, these analyses were limited by the modest number of studies, missing or inconsistently reported study-level covariates, and collinearity between publication era, geography, diagnostic strategy, and gatekeeping. Therefore, residual heterogeneity remains substantial, and pooled estimates should be interpreted as broad indicators of diagnostic yield rather than precise prevalence estimates. Although we explored publication era descriptively, robust meta-regression by era was limited by the very small number of pre-2015 studies and by strong confounding between era, clinical setting, diagnostic protocol, and verification strategy. Therefore, temporal trends in CA prevalence could not be separated reliably from changes in screening intensity and diagnostic sensitivity. The ‘no specific red flags’ estimate is especially limited by the small number of studies, profound design heterogeneity, and partial verification in two of the three cohorts; therefore, it should not be interpreted as a definitive population-level prevalence estimate. Pooled estimates from enriched clinical frames should not be extrapolated to the general population. Finally, subtype-specific estimates should also be interpreted cautiously because diagnostic work-up and amyloid typing were not uniform across studies; nevertheless, the observed differences in ATTR:AL ratios provide a useful signal for tailoring screening pathways to the clinical context.

In summary, CA remains uncommon in unselected populations but is not rare in several routine clinical settings, particularly HFpEF/HFmrEF, severe AS, and LVH/HCM, and is enriched in orthopaedic red-flag cohorts when cardiac findings coexist. These results justify targeted screening, capacity planning for confirmatory diagnostics, and careful stewardship of disease-modifying therapies, with the aim of diagnosing CA before irreversible disease progression has occurred, while motivating standardized diagnostic workflows and prospective all-comer studies designed to yield unbiased population estimates.

## Clinical perspectives

CA is not rare in routine high-yield settings, with pooled prevalence approaching or exceeding 10% in HFpEF/HFmrEF, severe AS, and LVH/HCM, and rising further among patients selected for confirmatory testing. These findings support a pragmatic stepwise screening approach. Clinicians should first identify high-risk contexts—older patients with HFpEF/HFmrEF, severe AS, unexplained LVH/HCM, or orthopaedic red flags such as bilateral CTS, lumbar spinal stenosis, or biceps tendon rupture. First-line assessment should include clinical red flags, ECG, echocardiography with wall thickness and, when available, strain imaging, plus natriuretic peptides/troponin; cardiac magnetic resonance may be used when echocardiography is inconclusive. Patients should proceed to confirmatory CA testing when unexplained LV wall thickness is present, particularly ≥12 mm, or when discordant ECG–echo findings, apical sparing, restrictive physiology, disproportionate biomarker elevation, conduction disease, atrial fibrillation, or extracardiac red flags increase suspicion. Confirmatory testing should combine bone scintigraphy with monoclonal protein assessment using serum free light chains and serum/urine immunofixation. Grades 2 and 3 cardiac uptake without monoclonal protein supports ATTR-CA, whereas any monoclonal abnormality or discordant result should prompt evaluation for AL amyloidosis and consideration of biopsy.

## Translational outlook

Future research should focus on prospective all-comer or randomly sampled cohorts in key clinical settings and in the general elderly population, ensuring that either all eligible participants or a predefined random sample undergo a full diagnostic work-up to yield unbiased prevalence estimates. Standardized diagnostic pathways and risk-based tools (including EHR-integrated triggers) need to be developed and validated to identify patients most likely to benefit from confirmatory testing while formally assessing the cost-effectiveness and operational impact of different screening strategies. Large registries and interventional studies should determine whether earlier detection via targeted screening, coupled with disease-modifying therapies, improves survival, functional status, and quality of life, and clarify optimal approaches to genetic counseling and long-term follow-up in variant ATTR-CA.

## Supplementary Material

xvag172_Supplementary_Data

## Declarations

### Data Availability

No data were generated or analysed for this manuscript.

### Pre-registered Clinical Trial Number

None supplied.
