# Supplementary material for: Prevalence of cardiac amyloidosis in screening studies: a systematic review and meta-analysis
Source: ESC Heart Fail. 2026 Jun 22;13(5):xvag172. doi: 10.1093/eschf/xvag172 (PMC13595223; doi:10.1093/eschf/xvag172)
Supplement: Supplementary file 1 [file xvag172_supplementary_data.docx]

**Supplementary material**

**Prevalence of cardiac amyloidosis in screening studies:**

**a systematic review and meta-analysis**

Alberto Aimo MD PhD^1,2^, Vincenzo Castiglione MD^1,2^, Giorgia Panichella MD^3^, Marianna Fontana MD PhD^3,^ Michele Emdin MD PhD^1,2^, Giuseppe Vergaro MD PhD^1,2^

1. Health Sciences Interdisciplinary Center, Scuola Superiore Sant’Anna, Pisa, Italy; 2. Cardiology Division, Fondazione Toscana Gabriele Monasterio, Pisa, Italy; 3. National Amyloidosis Centre, University College London, Royal Free Campus, London, United Kingdom.

**Search strategy**

PubMed/MEDLINE:

(prevalence[ti] OR frequency[ti] OR epidemiology[ti] OR screening[ti]) AND ("cardiac amyloidosis"[ti] OR amyloidosis[ti] OR transthyretin[ti] OR ATTR[ti] OR "light-chain amyloidosis"[ti] OR "light chain amyloidosis"[ti] OR "AL amyloidosis"[ti]) AND english[lang] AND ("0001/01/01"[Date - Publication] : "2025/08/10"[Date - Publication]) NOT (case reports[Publication Type])

EMBASE:

('prevalence':ti OR 'frequency':ti OR 'epidemiology':ti OR 'screening':ti) AND ('cardiac amyloidosis':ti OR amyloidosis:ti OR transthyretin:ti OR attr:ti OR 'light chain amyloidosis':ti OR 'al amyloidosis':ti) AND [english]/lim AND [humans]/lim NOT 'case report'/it

**Results**

### Exploratory analyses

Exploratory analyses by publication era showed that most available evidence came from the contemporary diagnostic era. Only four reports were published before 2015, and these were limited to HF, AS, and bone scintigraphy for non-cardiac indications; no pre-2015 studies were available for several key settings, including orthopedic disease, LVH/HCM, atrial fibrillation, peripheral neuropathy, or individuals without specific red flags. Therefore, within-setting comparisons between pre-2015 and 2015–2025 studies were not sufficiently powered for most settings. These analyses are reported in **Supplementary Table 4**.

Among settings with ≥3 studies, CA prevalence was high in cohorts grouped under LVH/HCM; however, this category was clinically heterogeneous and included both LVH/unexplained wall-thickening cohorts and one tentative HCM cohort. In a post-hoc descriptive analysis, three studies were classified as LVH/unexplained wall-thickening cohorts, whereas one study enrolled patients with a tentative HCM diagnosis. Because the HCM subgroup included only one study, it was not pooled separately.

### Study heterogeneity, risk of bias, and publication bias

Using a GRADE-based approach adapted for prevalence studies, we rated the certainty of the pooled estimates as generally low, with some variation by setting (**Supplementary Table 5**). Heterogeneity between studies was usually high (**Supplementary Table 6**). In most settings, removing individual studies one at a time changed the pooled prevalence by ≤1 percentage point, indicating that no single study dominated the results (**Supplementary Table 7**). Larger changes in LVH/HCM and in unselected populations reflected the small number of available studies. In settings with gatekeeping, prevalence calculated among tested participants was higher than in studies that tested all enrollees, whereas whole-cohort prevalence tended to be lower because many untested participants were implicitly counted as not having CA (**Supplementary Table 8**).

The risk of bias is reported in **Supplementary Table 9**. The risk was classified as high or moderate in 78 studies out of 82. Common reasons for moderate or high risk of bias included selection bias, partial verification or gatekeeping, and measurement indirectness, particularly imaging uptake used as a proxy without universal monoclonal testing.

In settings with ≥10 studies, Egger’s regression suggested small-study effects, while Begg’s test was significant only in the HF setting (**Supplementary Table 10**). Given the substantial heterogeneity and non-uniform verification across studies, these findings cannot distinguish publication bias from design-related asymmetry.

**Supplementary Table 1. Prevalence of cardiac amyloidosis (CA) in the selected studies.**

| **First author, year^ref^** | **Study setting** | **CA diagnosed, n** | **ATTR-CA, n** | **AL-CA, n** | **CA prevalence on the whole cohort (%)** | **ATTR-CA prevalence on the whole cohort (%)** | **AL-CA prevalence on the whole cohort (%)** | **CA prevalence in the subgroup undergoing further testing (%)** | **ATTR-CA prevalence in the subgroup undergoing further testing (%)** | **AL-CA prevalence in the subgroup undergoing further testing (%)** | **Notes** |
| --- | --- | --- | --- | --- | --- | --- | --- | --- | --- | --- | --- |
| Mohammed, 2014(3) | Heart failure | 21 | 18 | 1 | 19.3 | 16.5 | 0.9 | 19.3 | 16.5 | 1.8 | 2 with unknown amyloid type |
| González-López, 2015(4) | Heart failure | 16 | 16 | 0 | 13.3 | 13.3 | 0 | 13.3 | 13.3 | 0 | Perugini 2-3=ATTR-CA, biopsy at the discretion of physician |
| Bennani Smires, 2016(5) | Heart failure | 15 | 9 | 6 | 30.6 | 18.4 | 12.2 | 30.6 | 18.4 | 12.2 | 1 AL-CA "suspected" |
| Arvanitis, 2017(6) | Heart failure | 2 | 2 | 0 | 2.0 | 2.0 | 0 | 2.0 | 2.0 | 0 | Positive genetic testing in 5, who were further screened |
| Ton, 2017(7) | Heart failure | 73 | 33 | 36 | 28.2 | 12.7 | 13.9 | 28.2 | N/A | N/A |  |
| López-Sainz, 2019(8) | Heart failure | 3 | 3 | 0 | 10.7 | 10.7 | 0 | 10.7 | 10.7 | 0 | 28 patients with HF |
| Hahn, 2020(9) | Heart failure | 15 | 11 | 3 | 13.9 | 10.2 | 2.8 | 13.9 | 10.2 | 2.8 | 1 AA-CA |
| AbouEzzedine, 2021(10) | Heart failure | 18 | 18 | 0 | 6.3 | 6.3 | 0 | 6.3 | 6.3 | 0 |  |
| Devesa, 2021(11) | Heart failure | 3 | 0 | 0 | 5.2 | 0 | 0 | 5.2 | 5.2 | 0 |  |
| Goland, 2021(12) | Heart failure | 7 | 7 | 0 | 9.3 | 9.3 | 0 | 9.3 | 9.3 | 0 |  |
| Lindmark, 2021(13) | Heart failure | 13 | 13 | 0 | 15.1 | 15.1 | 0 | 15.1 | 15.1 | 0 | 25/174 including known ATTR-CA = 14% |
| Jaramillo-Hidalgo, 2023(14) | Heart failure | 15 | 15 | 0 | 30 | 30 | 0 | 30 | 30 | 0 | Perugini 2-3=ATTR-CA |
| Madhani, 2023(15) | Heart failure | 19 | 19 | 0 | 6.8 | 6.8 | 0 | 6.8 | 6.8 | 0 |  |
| Ruiz-Hueso, 2023(16) | Heart failure | 91 | 76 | 2 | 20.1 | 16.8 | 0.4 | 20.1 | 16.8 | 0.4 | 13 patients with inconclusive amyloid typing |
| Spaccavento, 2024(17) | Heart failure | 16 | 16 | 0 | 15.5 | 15.5 | 0 | 15.5 | 15.5 | 0 |  |
| Tubben, 2024(18) | Heart failure | 5 | 5 | 0 | 4.8 | 4.8 | 0 | 4.8 | 4.8 | 0 |  |
| Yun, 2024(19) | Heart failure | 56 | 56 | 0 | 17.8 | 17.8 | 0 | 17.8 | 17.8 | 0 |  |
| Achten, 2025(20) | Heart failure | 6 | 6 | 0 | 3.0 | 3.0 | 0 | 3.0 | 3.0 | 0 |  |
| Garcia-Pavia, 2025(21) | Heart failure | 65 | 65 | 0 | 16.8 | 16.8 | 0 | 16.8 | 16.8 | 0 |  |
| Healy, 2025(22) | Heart failure | 7 | 7 | 0 | 8.1 | 8.1 | 0 | 8.1 | 8.1 | 0 |  |
| Nietlispach, 2012(23) | Aortic stenosis | 5 | N/A | N/A | 25.0 | N/A | N/A | 25.0 | N/A | N/A |  |
| Longhi, 2016(24) | Aortic stenosis | 5 | 5 | 0 | 11.6 | 11.6 | 0 | 100 | 100 | 0 |  |
| Treibel, 2016(25) | Aortic stenosis | 6 | 6 | 0 | 4.1 | 4.1 | 0 | 4.1 | 4.1 | 0 |  |
| Castano, 2017(26) | Aortic stenosis | 24 | 100 | 0 | 15.9 | 66.2 | 0 | 15.9 | 15.9 | 0 |  |
| Cavalcante, 2017(27) | Aortic stenosis | 9 | N/A | N/A | 8.0 | 0 | 0 | N/A | N/A | N/A | CA diagnosis relying mostly on CMR alone |
| Nitsche, 2020(28) | Aortic stenosis | 16 | 15 | 1 | 8.4 | 7.9 | 0.5 | 8.4 | 7.9 | 0.5 |  |
| Oda 2020(29) | Aortic stenosis | 7 | 7 | 0 | 3.6 | 3.6 | 0 | 28.0 | 28.0 | 0 |  |
| Scully, 2020(30) | Aortic stenosis | 26 | 26 | 0 | 13.0 | 13.0 | 0 | 13.0 | 13.0 | 0 | AL amyloid "felt unlikely in all cases", "all genotyped patients to date (n= 12) were wild-type ATTR" |
| Nitsche, 2021(31) | Aortic stenosis | 48 | 47 | 1 | 11.8 | 11.5 | 0.2 | N/A | N/A | N/A |  |
| Rosenblum, 2021(32) | Aortic stenosis | 27 | 27 | 0 | 13.2 | 13.2 | 0 | 13.2 | 13.2 | 0 |  |
| Shimoni, 2021(33) | Aortic stenosis | 11 | 11 | 0 | 12.5 | 12.5 | 0 | 12.5 | 12.5 | 0 |  |
| Singal, 2021(34) | Aortic stenosis | 0 | 0 | 0 | 0 | 0 | 0 | 0 | 0 | 0 |  |
| Costa, 2024(35) | Aortic stenosis | 7 | 7 | 0 | 7.0 | 7.0 | 0 | 7.0 | 7.0 | 0 | Bone tracer uptake = ATTR-CA |
| Jakstaite, 2024(36) | Aortic stenosis | 6 | 6 | 0 | 7.1 | 7.1 | 0 | 7.1 | 7.1 | 0 |  |
| SadrAldin, 2024(37) | Aortic stenosis | 5 | 5 | 0 | 18.5 | 18.5 | 0 | 18.5 | 18.5 | 0 |  |
| McShane, 2025(38) | Aortic stenosis | 2 | 2 | 0 | 1.2 | 1.2 | 0 | 6.7 | 6.7 | 0 |  |
| Sperry, 2018(39) | Orthopedic disease | 2 | 1 | 1 | 2.0 | 1.0 | 1.0 | N/A | N/A | N/A |  |
| Zegri-Reiriz, 2019(40) | Orthopedic disease | 3 | 2 | 1 | 1.3 | 0.9 | 0.4 | 3.0 | 2.0 | 1.0 | CTS alone: 1.2%. LVH+bilateral CTS: 5.5%. |
| Sperry, 2021(41) | Orthopedic disease | 0 | 0 | 0 | 0 | 0 | 0 | 0 | 0 | 0 |  |
| Sugiura, 2021(42) | Orthopedic disease | 3 | 3 | 0 | 3.8 | 3.8 | 0 | 18.8 | 18.8 | 0 |  |
| Vianello, 2021(43) | Orthopedic disease | 2 | 2 | 0 | 3.8 | 3.8 | 0 | 33.0 | 33.0 | 0 | 2/6 patients: no AL, declined scintigraphy |
| Baylor, 2022(44) | Orthopedic disease | 0 | 0 | 0 | 0 | 0 | 0 | 0 | 0 | 0 |  |
| Westin, 2022(45) | Orthopedic disease | 12 | 12 | 0 | 4.8 | 4.8 | 0 | 4.8 | 4.8 | 0 |  |
| Debonnaire, 2023(46) | Orthopedic disease | 3 | 3 | 0 | 3.7 | 3.7 | 0 | 3.7 | 3.7 | 0 |  |
| Takashio, 2023(47) | Orthopedic disease | 6 | 6 | 0 | 5.0 | 5.0 | 0 | 50 | 50 | 0 |  |
| Andrei, 2024(48) | Orthopedic disease | 0 | 0 | 0 | 0 | 0 | 0 | 0 | 0 | 0 |  |
| Gannon, 2024(49) | Orthopedic disease | 1 | 1 | 0 | 1.6 | 1.6 | 0 | 7.1 | 7.1 | 0 |  |
| Yamada, 2024(50) | Orthopedic disease | 2 | 2 | 0 | 4.9 | 4.9 | 0 | 4.9 | 4.9 | 0 |  |
| Eldhagen, 2025(51) | Orthopedic disease | 3 | 3 | 0 | 15.8 | 15.8 | 0 | 15.8 | 15.8 | 0 |  |
| Pimenta, 2025(52) | Orthopedic disease | 4 | 4 | 0 | 18.2 | 18.2 | 0 | 18.2 | 18.2 | 0 |  |
| al-Nahhas, 1995(53) | Bone scintigraphy for non-cardiac reasons | 0 | 0 | 0 | 0 | 0 | 0 | 0 | 0 | 0 | Increased cardiac uptake in 18 patients, but no evidence of cardiac disease |
| Longhi, 2014(54) | Bone scintigraphy for non-cardiac reasons | 14 | N/A | N/A | 0.1 | N/A | N/A | 0.1 | N/A | N/A | 0.36% with cardiac uptake, 14/32 agreed to further testing |
| Mohamed-Salem, 2018(55) | Bone scintigraphy for non-cardiac reasons | 31 | 31 | 0 | 2.8 | 2.8 | 0 | 2.8 | 2.8 | 0 | Perugini 2-3=ATTR-CA |
| Kim, 2019(56) | Bone scintigraphy for non-cardiac reasons | 4 | N/A | N/A | 0 | N/A | N/A | 0 | N/A | N/A | Other 2 subjects without LVH. Positive DPD=ATTR-CA |
| Bianco, 2021(57) | Bone scintigraphy for non-cardiac reasons | 23 | N/A | N/A | 0.5 | N/A | N/A | 0.5 | N/A | N/A | Perugini 2-3=ATTR-CA |
| Cuscaden, 2021(58) | Bone scintigraphy for non-cardiac reasons | 15 | 15 | 0 | 0.4 | 0.4 | 0 | 0.4 | 0.4 | 0 | HMDP positivity=ATTR-CA; MDP scans not considered (currently not recommended) |
| Halme, 2022(59) | Bone scintigraphy for non-cardiac reasons | 47 | 47 | 0 | 3.5 | 3.5 | 0 | 3.5 | 3.5 | 0 | Perugini 2-3=ATTR-CA |
| Nitsche, 2022(60) | Bone scintigraphy for non-cardiac reasons | N/A | N/A | N/A | N/A | N/A | N/A | 1.7 | N/A | N/A | Perugini 2-3=ATTR-CA; prevalence calculated on non-cardiac referrals (90.6% of the whole cohort) |
| Salvalaggio, 2022(61) | Bone scintigraphy for non-cardiac reasons | 20 | 20 | 0 | 0.2 | 0.2 | 0 | 2.1 | 2.1 | 0 | Number of scans reported, not of patients; Perugini 2-3=ATTR-CA |
| Suomalainen, 2022(62) | Bone scintigraphy for non-cardiac reasons | 69 | 69 | 0 | 3.5 | 3.5 | 0 | 3.5 | 3.5 | 0 | Perugini 2-3=ATTR-CA |
| Navarro-Saez, 2023(63) | Bone scintigraphy for non-cardiac reasons | N/A | N/A | N/A | N/A | N/A | N/A | 1.2 | N/A | N/A | Subgroup with no cardiac indication to scan; myocardial uptake assimilated to ATTR-CA |
| Son, 2024(64) | Bone scintigraphy for non-cardiac reasons | N/A | N/A | N/A | N/A | N/A | N/A | 0 | 0 | N/A | Possible ATTR-CA assimilated to ATTR-CA. Possible ATTR-CA: n=14; only 5 completed the diagnostic workup |
| Cariou, 2017(65) | LVH/HCM | 50 | 31 | 18 | 43.9 | 27.2 | 15.8 | 43.9 | 27.2 | 15.8 | 1 AA-CA |
| Maurizi, 2020(66) | LVH/HCM | 32 | 28 | 3 | 9.3 | 8.2 | 0.9 | 9.3 | 8.2 | 0.9 | 11 vATTR, 17 wtATTR |
| Nakao, 2021(67) | LVH/HCM | 54 | 23 | 22 | 18.3 | 7.8 | 7.5 | 18.3 | 7.8 | 7.5 |  |
| Merlo, 2022(68) | LVH/HCM | 62 | 51 | 11 | 5.3 | 4.4 | 0.9 | 6.2 | 5.1 | 1.1 | 1.2% of unselected echos |
| Remior-Pérez, 2025(69) | Atrial fibrillation | 10 | 9 | 0 | 8.3 | 7.4 | 0 | 8.3 | 7.4 | 0 | 5 with definite ATTR-CA, 4 with likely ATTR-CA |
| Yamasaki, 2024(70) | Atrial fibrillation | 6 | 6 | 0 | 1.8 | 1.8 | 0 | 1.8 | 1.8 | 0 |  |
| Shinzato, 2025(71) | Atrial fibrillation | 13 | 11 | 2 | 3.4 | 2.9 | 0.5 | 3.4 | 2.9 | 0.5 | Only subgroup undergoing RV biopsy considered (other patients underwent atrial, but not RV biopsy) |
| Porcari, 2021(72) | No specific red flag | 24 | 12 | 12 | 42.9 | 21.4 | 21.4 | 43.0 | 21.0 | 21.0 |  |
| Aimo, 2024(73) | No specific red flag | 4 | 4 | 0 | 0.4 | 0.4 | 0 | 1.9 | 1.9 | 0 |  |
| Arima, 2025(74) | No specific red flag | 2 | 2 | 0 | 0.2 | 0.2 | 0 | 0.2 | 0.2 | 0 | 22 patients with elevated troponin refusing further exams, but computed in the prevalence |
| Di Stefano, 2024(75) | Peripheral neuropathy | 5 | 5 | 0 | 3.4 | 3.4 | 0 | 3.5 | 3.5 | 0 |  |
| Doneddu, 2025(76) | Peripheral neuropathy | 0 | 0 | 0 | 0 | 0 | 0 | 0 | 0 | 0 |  |
| Samuelsson, 2019(77) | Peripheral neuropathy | 0 | 0 | 0 | 0 | 0 | 0 | 0 | 0 | 0 |  |
| de Frutos, 2024(78) | One or more red flags | 0 | 0 | 0 | 0 | 0 | 0 | 0 | 0 | 0 |  |
| Di Stefano, 2025(79) | One or more red flags | 0 | 0 | 0 | 0 | 0 | 0 | 0 | 0 | 0 |  |
| Aimo, 2025(1) | Two red flags | 343 | 311 | 32 | 28.2 | 25.6 | 2.6 | 28.2 | 25.6 | 2.6 |  |
| López-Sainz, 2019(8) | Conduction disorders | 2 | 2 | 0 | 1.8 | 1.8 | 0 | 1.8 | 1.8 | 0 |  |
| Cannie, 2024(80) | Conduction disorders | 3 | 3 | 0 | 7.7 | 7.7 | 0 | 7.7 | 7.7 | 0 |  |
| Mangiacavalli, 2025(81) | Plasma cell disorders | 22 | 0 | 22 | 1.6 | 0 | 1.6 | 1.6 | 0 | 1.6 |  |
| Zhou, 2025(82) | Plasma cell disorders | 3 | 0 | 3 | 10 | 0 | 10 | 10 | 0 | 10 |  |
| Quarta, 2015(83) | Other specific setting | 5 | 5 | 0 | 0.4 | 0.4 | 0 | 0.4 | 0.4 | 0 | Subgroup with available echo considered; 5 patients with Val122Ile and LV wall thickness ≥12 mm, but only 3 with severe symmetric LV wall thickening and features suggestive of an infiltrative phenotype |
| Tebbe, 2016(84) | Other specific setting | 6 | N/A | N/A | 10.5 | N/A | N/A | 10.5 | N/A | N/A |  |

AA, amyloid A, AL, light-chain amyloid; ATTR, amyloid transthyretin; CTS, carpal tunnel syndrome; HF, heart failure; HMDP, hydroxymethylene diphosphonate; LV, left ventricular; LVH, left ventricular hypertrophy; MDP, methylene diphosphonate; N/A, not available; RV right ventricular; v, variant; wt, wild-type.

**Supplementary Table 2. Men:women (M:W) ratio among patients diagnosed with cardiac amyloidosis (CA).**

| **Setting** | **M:W ratio (95% CI)** |
| --- | --- |
| HF | 2.10 (1.55-2.83) |
| Aortic stenosis | 2.77 (1.71-4.49) |
| Orthopedic disease | 2.90 (1.24-6.75) |
| Bone scintigraphy for non-cardiac reasons | 4.05 (2.76-5.94) |
| LV hypertrophy/HCM | 2.61 (1.16-5.88) |
| No specific red flag | 1.09 (0.53-2.25) |

Heterogeneity metrics could not be calculated for the peripheral neuropathy and atrial fibrillation settings because of the lack of information from at least 3 studies. CI, confidence interval; HCM, hypertrophic cardiomyopathy; HF, heart failure; LV, left ventricular.

**Supplementary Table 3. Ratio between wild-type (wt) and variant (v) disease among patients diagnosed with cardiac amyloidosis (CA).**

| **Setting** | **wt:v ratio (95% CI)** |
| --- | --- |
| HF | 5.72 (3.16-10.35) |
| Aortic stenosis | 26.35 (8.26-84.02) |
| Orthopedic disease | 6.94 (2.81-17.12) |
| LVH/HCM | 2.73 (0.83-8.96) |
| Atrial fibrillation | 15.75 (2.08-119.01) |
| No specific red flag | 6.78 (0.83-55.70) |

Heterogeneity metrics could not be calculated for the peripheral neuropathy and bone scintigraphy settings because of the lack of information from at least 3 studies. CI, confidence interval; HCM, hypertrophic cardiomyopathy; HF, heart failure; LVH, left ventricular hypertrophy.

**Supplementary Table 4. Prevalence of cardiac amyloidosis according to publication era and clinical setting.**

| **Setting** | **Denominator** | **Era** | **Studies, n** | **Pooled prevalence or range** | **Notes** |
| --- | --- | --- | --- | --- | --- |
| HF | Whole cohort | pre-2015 | 1 | descriptive only | insufficient for pooling |
| HF | Whole cohort | 2015–2025 | 19 | pooled estimate | contemporary diagnostic era |
| AS | Whole cohort | pre-2015 | 1 | descriptive only | insufficient for pooling |
| AS | Whole cohort | 2015–2025 | 15 | pooled estimate | contemporary diagnostic era |
| Bone scintigraphy | Whole cohort | pre-2015 | 2 | descriptive only | older scintigraphy era |
| Bone scintigraphy | Whole cohort | 2015–2025 | 10 | pooled estimate | contemporary imaging era |

Abbreviations: AS, aortic stenosis; CA, cardiac amyloidosis; HF, heart failure.

**Supplementary Table 5. Grading of the** **certainty (confidence)** **of the evidence for each pooled prevalence outcome.**

| **Setting** | **Denominator** | **k** | **Total N** | **Total diagnoses** | **Pooled prev (%)** | **95% CI low (%)** | **95% CI high (%)** | **95% PI low (%)** | **95% PI high (%)** | **I^2 (%)** | **Tau^2** | **Gatekeeping share** | **Certainty** | **Reasons** |
| --- | --- | --- | --- | --- | --- | --- | --- | --- | --- | --- | --- | --- | --- | --- |
| HF | Whole cohort | 20 | 3255 | 466 | 12.64 | 9.07 | 17.34 | 3.96 | 33.65 | 85.7 | 0.33447 | 0.05 | Very low | Inconsistency: very high heterogeneity (I²=86%); Imprecision: 95% CI crosses decision-relevant band(s); Publication bias: small-study effects possible (k≥10) |
|  | Further testing for CA | 20 | 3161 | 466 | 13.58 | 9.95 | 18.27 | 4.48 | 34.53 | 84.7 | 0.31091 | 0.05 | Very low | Inconsistency: substantial heterogeneity (I²=85%); Indirectness: tested-only denominator (not all enrollees); Imprecision: 95% CI crosses decision-relevant band(s); Publication bias: small-study effects possible (k≥10) |
| Aortic stenosis | Whole cohort | 16 | 2160 | 204 | 9.54 | 6.79 | 13.25 | 3.82 | 21.89 | 66.5 | 0.18696 | 0.19 | Very low | Inconsistency: substantial heterogeneity (I²=66%); Imprecision: 95% CI crosses decision-relevant band(s); Publication bias: small-study effects possible (k≥10) |
|  | Further testing for CA | 16 | 1824 | 204 | 11.9 | 8.35 | 16.7 | 4.95 | 25.93 | 64.4 | 0.17645 | 0.19 | Very low | Inconsistency: substantial heterogeneity (I²=64%); Indirectness: tested-only denominator (not all enrollees); Imprecision: 95% CI crosses decision-relevant band(s); Publication bias: small-study effects possible (k≥10) |
| Orthopedic disease | Whole cohort | 14 | 1246 | 41 | 4.07 | 2.4 | 6.82 | 1.05 | 14.48 | 47.8 | 0.34493 | 0.64 | Very low | Risk of bias: frequent partial verification (≥50% gated); Imprecision: 95% CI crosses decision-relevant band(s); Publication bias: small-study effects possible (k≥10) |
|  | Further testing for CA | 14 | 606 | 41 | 12.59 | 6.19 | 23.91 | 1.24 | 62.23 | 71.5 | 1.12142 | 0.64 | Very low | Risk of bias: frequent partial verification (≥50% gated); Inconsistency: substantial heterogeneity (I²=72%); Indirectness: tested-only denominator (not all enrollees); Imprecision: 95% CI crosses decision-relevant band(s); Publication bias: small-study effects possible (k≥10) |
| Bone scintigraphy for non-cardiac reasons | Whole cohort | 9 | 44710 | 223 | 0.49 | 0.13 | 1.83 | 0.01 | 15.09 | 97.8 | 2.04226 | 0 | Very low | Inconsistency: very high heterogeneity (I²=98%); Imprecision: 95% CI crosses decision-relevant band(s) |
|  | Further testing for CA | 9 | 44710 | 223 | 0.49 | 0.13 | 1.83 | 0.01 | 15.09 | 97.8 | 2.04226 | 0 | Very low | Inconsistency: very high heterogeneity (I²=98%); Indirectness: tested-only denominator (not all enrollees); Imprecision: 95% CI crosses decision-relevant band(s) |
| LVH/HCM | Whole cohort | 4 | 1921 | 198 | 15.08 | 2.83 | 51.99 | 0.09 | 97.33 | 97.9 | 1.22 | 0.5 | Very low | Risk of bias: frequent partial verification (≥50% gated); Inconsistency: very high heterogeneity (I²=98%); Imprecision: 95% CI crosses decision-relevant band(s) |
|  | Further testing for CA | 4 | 652 | 198 | 40.38 | 5.3 | 89.12 | 1.65 | 96.47 | 93.7 | 0.56756 | 0.5 | Very low | Risk of bias: frequent partial verification (≥50% gated); Inconsistency: very high heterogeneity (I²=94%); Indirectness: tested-only denominator (not all enrollees); Imprecision: 95% CI crosses decision-relevant band(s) |
| Atrial fibrillation | Whole cohort | 3 | 848 | 29 | 3.77 | 0.53 | 22.28 | 0 | 99.91 | 80.1 | 0.45839 | 0.33 | Low | Inconsistency: substantial heterogeneity (I²=80%); Imprecision: 95% CI crosses decision-relevant band(s) |
|  | Further testing for CA | 3 | 575 | 29 | 6.04 | 1.51 | 21.21 | 0 | 99.36 | 68.5 | 0.25232 | 0.33 | Very low | Inconsistency: substantial heterogeneity (I²=68%); Indirectness: tested-only denominator (not all enrollees); Imprecision: 95% CI crosses decision-relevant band(s) |
| No specific red flag | Whole cohort | 3 | 2197 | 30 | 1.76 | 0 | 98.47 | 0 | 100 | 98.4 | 13.69847 | 0.67 | Very low | Risk of bias: frequent partial verification (≥50% gated); Inconsistency: very high heterogeneity (I²=98%); Imprecision: 95% CI crosses decision-relevant band(s) |
|  | Further testing for CA | 3 | 327 | 30 | 7.76 | 0.06 | 91.98 | 0 | 100 | 96 | 5.29884 | 0.67 | Very low | Risk of bias: frequent partial verification (≥50% gated); Inconsistency: very high heterogeneity (I²=96%); Indirectness: tested-only denominator (not all enrollees); Imprecision: 95% CI crosses decision-relevant band(s) |
| Peripheral neuropathy | Whole cohort | 3 | 424 | 5 | 1.17 | 0.04 | 28.07 | 0 | 100 | 54.2 | 1.36323 | 0.33 | Moderate | Imprecision: 95% CI crosses decision-relevant band(s) |
|  | Further testing for CA | 3 | 381 | 5 | 1.4 | 0.06 | 26.18 | 0 | 100 | 45.8 | 0.97457 | 0.33 | Low | Indirectness: tested-only denominator (not all enrollees); Imprecision: 95% CI crosses decision-relevant band(s) |

CA, cardiac amyloidosis; CI, confidence interval; HCM, hypertrophic cardiomyopathy; HF, heart failure; LVH, left ventricular hypertrophy; PI, prediction interval.

**Supplementary Table 6. Heterogeneity between studies in the prevalence estimates of cardiac amyloidosis (CA).**

| **Setting** | **Whole cohorts** | | | **Further testing for CA** | | |
| --- | --- | --- | --- | --- | --- | --- |
|  | **I^2^** | **tau^2^** | **Q** | **I^2^** | **tau^2^** | **Q** |
| HF | 85.7 | 0.08 | 132.84 | 85.7 | 0.08 | 132.84 |
| Aortic stenosis | 67.2 | 0.05 | 42.65 | 65.2 | 0.05 | 34.47 |
| Orthopedic disease | 50.7 | 0.08 | 20.28 | 75.7 | 26.05 | 37.06 |
| Bone scintigraphy for non-cardiac reasons | 98.1 | 0.51 | 366.57 | 97.7 | 0.41 | 298.75 |
| LVH/HCM | 97.9 | 0.31 | 144.51 | 97.6 | 0.27 | 127.09 |
| Atrial fibrillation | 80.1 | 0.11 | 10.07 | 79.5 | 0.11 | 9.75 |
| No specific red flag | 98.4 | 3.42 | 127.16 | 97.8 | 2.42 | 89.64 |

Heterogeneity metrics could not be calculated for the peripheral neuropathy setting. HCM, hypertrophic cardiomyopathy; HF, heart failure; LVH, left ventricular hypertrophy.

**Supplementary Table 7. Influence of single studies on the estimated prevalence of cardiac amyloidosis (CA): leave-one-out analysis.**

| **Setting** | **Denominator** | **Max \|Δ\| (pp)** | **Median \|Δ\| (pp)** |
| --- | --- | --- | --- |
| HF | Whole cohort | 0.931 | 0.390 |
|  | Further testing for CA | 0.970 | 0.322 |
| Aortic stenosis | Whole cohort | 0.722 | 0.332 |
|  | Further testing for CA | 0.719 | 0.347 |
| Orthopedic disease | Whole cohort | 0.557 | 0.172 |
|  | Further testing for CA | 2.29 | 0.734 |
| Bone scintigraphy for non-cardiac reasons | Whole cohort | 0.179 | 0.109 |
|  | Further testing for CA | 0.179 | 0.109 |
| LVH/HCM | Whole cohort | 5.674 | 3.878 |
|  | Further testing for CA | 11.795 | 10.958 |
| Atrial fibrillation | Whole cohort | 1.462 | 1.171 |
|  | Further testing for CA | 2.382 | 0.846 |
| No specific red flag | Whole cohort | 3.524 | 1.860 |
|  | Further testing for CA | 7.581 | 5.448 |
| Peripheral neuropathy | Whole cohort | 0.808 | 0.610 |
|  | Further testing for CA | 1.010 | 0.959 |

Maximum and minimum percent point (pp) changes in the estimated prevalence of CA by removing one study at the time from each setting are reported. HF, heart failure; LVH, left ventricular hypertrophy.

**Supplementary Table 8. Changes in the pooled prevalence of cardiac amyloidosis (CA) according to gatekeeping.**

| **Setting** | **Denominator** | **Gatekeeping** | **k** | **Pooled prevalence (%)** | **95% CI low (%)** | **95% CI high (%)** |
| --- | --- | --- | --- | --- | --- | --- |
| Heart failure | Whole cohort | No gatekeeping | 19 | 13.27 | 9.7 | 17.89 |
|  | Further testing for CA | No gatekeeping | 19 | 13.27 | 9.7 | 17.89 |
| Aortic stenosis | Whole cohort | Gatekeeping present | 3 | 4.14 | 0.26 | 41.92 |
|  | Whole cohort | No gatekeeping | 13 | 10.95 | 8.32 | 14.29 |
|  | Further testing for CA | Gatekeeping present | 3 | 28.58 | 0.46 | 97.18 |
|  | Further testing for CA | No gatekeeping | 13 | 10.95 | 8.32 | 14.29 |
| Orthopedic disease | Whole cohort | Gatekeeping present | 9 | 3 | 1.85 | 4.83 |
|  | Whole cohort | No gatekeeping | 5 | 6.99 | 1.95 | 22.11 |
|  | Further testing for CA | Gatekeeping present | 9 | 19.12 | 7.82 | 39.7 |
|  | Further testing for CA | No gatekeeping | 5 | 6.99 | 1.95 | 22.11 |
| Bone scintigraphy for non-cardiac reasons | Whole cohort | No gatekeeping | 9 | 0.49 | 0.13 | 1.83 |
|  | Further testing for CA | No gatekeeping | 9 | 0.49 | 0.13 | 1.83 |
| LVH/HCM | Whole cohort | Gatekeeping present | 2 | 6.96 | 0.16 | 78.03 |
|  | Whole cohort | No gatekeeping | 2 | 29.39 | 0.01 | 99.91 |
|  | Further testing for CA | Gatekeeping present | 2 | 70.42 | 0 | 100 |
|  | Further testing for CA | No gatekeeping | 2 | 29.39 | 0.01 | 99.91 |
| Atrial fibrillation | Further testing for CA | No gatekeeping | 2 | 5.24 | 0.01 | 95.76 |
|  | Whole cohort | No gatekeeping | 2 | 5.24 | 0.01 | 95.76 |
| No specific red flag | Whole cohort | Gatekeeping present | 2 | 0.3 | 0 | 30.23 |
|  | Further testing for CA | Gatekeeping present | 2 | 2.32 | 0.04 | 59.79 |
| Peripheral neuropathy | Whole cohort | No gatekeeping | 2 | 1.53 | 0 | 100 |
|  | Further testing for CA | No gatekeeping | 2 | 1.53 | 0 | 100 |
| Conduction disorders | Whole cohort | No gatekeeping | 2 | 3.94 | 0 | 99.83 |
|  | Further testing for CA | No gatekeeping | 2 | 3.94 | 0 | 99.83 |
| Plasma cell disorders | Whole cohort | No gatekeeping | 2 | 3.76 | 0 | 99.99 |
|  | Further testing for CA | No gatekeeping | 2 | 3.76 | 0 | 99.99 |
| One or more red flags | Whole cohort | No gatekeeping | 2 | 0.57 | 0 | 99.98 |
|  | Further testing for CA | No gatekeeping | 2 | 0.57 | 0 | 99.98 |
|  | Whole cohort | No gatekeeping | 2 | 2.14 | 0 | 100 |
|  | Further testing for CA | No gatekeeping | 2 | 2.14 | 0 | 100 |

Gatekeeping consists in the testing for CA only in a subgroup of enrolled subjects who meet specific additional criteria. HF, heart failure; LVH, left ventricular hypertrophy.

**Supplementary Table 9. Risk of bias assessment.**

| **Study** | **Selection bias** | **Verification/**  **attrition bias** | **Case ascertainment** | **Measurement consistency** | **Overall risk** | **Q1 Sample frame appropriate?** | **Q2 Participants sampled appropriately?** | **Q3 Sample size adequate?** | **Q4 Subjects and setting described in detail?** | **Q5 Data analysis covered identified sample?** | **Q6 Valid methods used to identify condition?** | **Q7 Condition measured in a standard, reliable way for all participants?** | **Q8 Appropriate statistical analysis?** | **Q9 Response rate adequate and managed?** | **JBI overall risk** |
| --- | --- | --- | --- | --- | --- | --- | --- | --- | --- | --- | --- | --- | --- | --- | --- |
| AbouEzzedine 2021 | Low | Low | Low | Low | Low–Moderate | Yes | Yes | Unclear | Yes | Yes | Yes | Yes | Yes | Yes | Low |
| Achten 2025 | Moderate | Low | Low | Low | Moderate | Unclear | Unclear | Unclear | Yes | Yes | Yes | Yes | Yes | Yes | Moderate |
| Aimo 2024 | Moderate–High | High | Low | Moderate | Moderate–High | No | No | Unclear | Yes | No | Yes | Unclear | Yes | No | High |
| Aimo 2025 | Moderate–High | Low | Low | Low | Moderate | No | Unclear | Yes | Yes | Yes | Yes | Yes | Yes | No | Moderate |
| Al-Nahhas 1994 | Moderate | Moderate | Moderate–High | Moderate | High | Unclear | Unclear | Unclear | Yes | Unclear | No | Unclear | Unclear | Unclear | High |
| Andrei 2024 | High | High | Low | Moderate | High | No | No | Unclear | Yes | No | Yes | Unclear | Yes | No | High |
| Arima 2025 | Moderate–High | High | Low | Moderate | Moderate–High | No | No | Unclear | Yes | No | Yes | Unclear | Yes | No | High |
| Arvanitis 2017 |  |  |  |  | Moderate–High | No | No | Unclear | Yes | Unclear | Unclear | Unclear | Yes | Unclear | High |
| Baylor 2022 |  |  |  |  | High (for CA prevalence) | No | Unclear | No | Yes | Yes | Yes | Yes | Yes | Yes | Moderate |
| Bennani Smires 2016 |  |  |  |  | Moderate–High | No | Unclear | Unclear | Yes | No | Yes | Unclear | Yes | Unclear | High |
| Bianco 2021 | Low–Moderate | Moderate | Moderate | Moderate | Moderate | Yes | Yes | Unclear | Yes | Unclear | Unclear | Unclear | Yes | Unclear | Moderate |
| Cannie 2024 | Moderate–High | Moderate | Moderate | Moderate | Moderate–High | No | No | Unclear | Yes | Unclear | Unclear | Unclear | Yes | Unclear | High |
| Cariou 2017 |  |  |  |  | Not directly applicable | No | Unclear | Unclear | Yes | Unclear | Yes | Yes | Yes | Unclear | Moderate |
| Castaño 2017 | Moderate | Low | Low | Low | Low–Moderate | Unclear | Unclear | Unclear | Yes | Yes | Yes | Yes | Yes | Yes | Moderate |
| Cavalcante 2017 | High | Moderate | Moderate–High | Moderate | High | No | No | Unclear | Yes | Unclear | No | Unclear | Unclear | Unclear | High |
| Costa 2024 | Moderate | Low | Low | Low | Low–Moderate | Unclear | Unclear | Unclear | Yes | Yes | Yes | Yes | Yes | Yes | Moderate |
| Cuscaden 2021 | Low–Moderate | Moderate | Moderate | Low–Moderate | Moderate | Yes | Yes | Unclear | Yes | Unclear | Unclear | Yes | Yes | Unclear | Moderate |
| de Frutos 2024 | Moderate | Low | Low | Low | Moderate (indirect to CA) | Unclear | Unclear | Unclear | Yes | Yes | Yes | Yes | Yes | Yes | Moderate |
| Debonnaire 2023 | Moderate | Low | Low | Low | Low–Moderate | Unclear | Unclear | Unclear | Yes | Yes | Yes | Yes | Yes | Yes | Moderate |
| Devesa 2021 | Moderate | High | Low | Moderate | Moderate | Unclear | Unclear | Unclear | Yes | No | Yes | Unclear | Yes | No | High |
| Di Stefano 2023 |  |  |  |  | Moderate–High (indirect to CA) | No | No | Unclear | Yes | Yes | Yes | Yes | Yes | Yes | Moderate |
| Di Stefano 2025 |  |  |  |  | Moderate–High (indirect to CA) | No | Unclear | No | Yes | Unclear | Yes | Yes | Yes | Unclear | Moderate |
| Doneddu 2025 | Moderate | Low | Low | Moderate | Moderate | Unclear | Unclear | Unclear | Yes | Yes | Yes | Unclear | Yes | Yes | Moderate |
| Eldhagen 2025 | High | Moderate | Low | Moderate | Moderate–High | No | No | Unclear | Yes | Unclear | Yes | Unclear | Yes | Unclear | High |
| Gannon 2024 | Moderate–High | Moderate | Low | Moderate | Moderate–High | No | No | Unclear | Yes | Unclear | Yes | Unclear | Yes | Unclear | High |
| García-Pavía 2025 | Low–Moderate | Low–Moderate | Low | Low–Moderate | Low–Moderate | Yes | Yes | Yes | Yes | Yes | Yes | Yes | Yes | Yes | Low |
| Goland 2021 | Moderate | Low | Low | Low–Moderate | Moderate | Unclear | Unclear | Unclear | Yes | Yes | Yes | Yes | Yes | Yes | Moderate |
| González-López 2015 | Low–Moderate | Moderate | Low | Low–Moderate | Low–Moderate | Yes | Yes | Unclear | Yes | Unclear | Yes | Yes | Yes | Unclear | Moderate |
| Hahn 2020 | Moderate | Low | Low | Low | Moderate | Unclear | Unclear | Unclear | Yes | Yes | Yes | Yes | Yes | Yes | Moderate |
| Halme 2022 | Low–Moderate | Moderate | Moderate–High | Moderate | Moderate–High | Yes | Yes | Unclear | Yes | Unclear | No | Unclear | Unclear | Unclear | High |
| Healy 2025 | Moderate | Low | Low | Low | Moderate | Unclear | Unclear | Unclear | Yes | Yes | Yes | Yes | Yes | Yes | Moderate |
| Jakstaite 2024 | Moderate–High | High | Low | Moderate | Moderate–High | No | No | Unclear | Yes | No | Yes | Unclear | Yes | No | High |
| Jaramillo-Hidalgo 2023 | Moderate | Moderate | Moderate | Moderate | Moderate | Unclear | Unclear | Unclear | Yes | Unclear | Unclear | Unclear | Yes | Unclear | Moderate |
| Kim 2019 | Low–Moderate | Moderate | Moderate | Moderate | Moderate | Yes | Yes | Yes | Yes | Unclear | Unclear | Unclear | Yes | Unclear | Moderate |
| Lindmark 2021 | Moderate | High | Low | Moderate | Moderate | Unclear | Unclear | Unclear | Yes | No | Yes | Unclear | Yes | No | High |
| Longhi 2014 | Moderate | Moderate | Moderate–High | Moderate | Moderate–High | Unclear | Unclear | Unclear | Yes | Unclear | No | Unclear | Unclear | Unclear | High |
| Longhi 2016 | High | Moderate | Moderate | Moderate | High | No | No | Unclear | Yes | Unclear | Unclear | Unclear | Yes | Unclear | High |
| Lopez-Sainz 2019 | Moderate | Moderate | Low | Moderate | Moderate | Unclear | Unclear | Unclear | Yes | Unclear | Yes | Unclear | Yes | Unclear | Moderate |
| Madhani 2023 | Moderate | Low | Low | Low | Low–Moderate | Unclear | Unclear | Unclear | Yes | Yes | Yes | Yes | Yes | Yes | Moderate |
| Mangiacavalli 2024 | High (for CA prevalence) | Moderate | Low | Moderate | Moderate (indirect to CA) | No | No | Unclear | Yes | Unclear | Yes | Unclear | Yes | Unclear | High |
| Maurizi 2019 | Moderate | Low | Low | Moderate | Moderate | Unclear | Unclear | Unclear | Yes | Yes | Yes | Unclear | Yes | Yes | Moderate |
| McShane 2025 | Moderate | Moderate | Low | Low | Low–Moderate | Unclear | Unclear | Unclear | Yes | Unclear | Yes | Yes | Yes | Unclear | Moderate |
| Merlo 2022 | Moderate–High | High | Low | Moderate | Moderate–High | No | No | Unclear | Yes | No | Yes | Unclear | Yes | No | High |
| Mohamed-Salem 2018 | Moderate | Moderate | Moderate | Moderate | Moderate | Unclear | Unclear | Unclear | Yes | Unclear | Unclear | Unclear | Yes | Unclear | Moderate |
| Mohammed 2014 | High | Low | Low | Low | Moderate (selection) | No | No | Unclear | Yes | Yes | Yes | Yes | Yes | Yes | High |
| Nakao 2021 |  |  | Low | Moderate | Not directly applicable | Unclear | Unclear | Unclear | Yes | Unclear | Yes | Unclear | Yes | Unclear | Moderate |
| Navarro-Saez 2023 | Moderate | Moderate | Moderate | Moderate | Moderate | Unclear | Unclear | Unclear | Yes | Unclear | Unclear | Unclear | Yes | Unclear | Moderate |
| Nietlispach 2012 | High | Low | Low | Moderate | High | No | No | Unclear | Yes | Yes | Yes | Unclear | Yes | Yes | High |
| Nitsche 2020 | Moderate | Low | Low | Low | Low–Moderate | Unclear | Yes | Unclear | Yes | Yes | Yes | Yes | Yes | Yes | Low |
| Nitsche 2021 | Moderate | Low | Low | Low | Low–Moderate | Unclear | Unclear | Unclear | Yes | Yes | Yes | Yes | Yes | Yes | Moderate |
| Nitsche 2022 | Low–Moderate | Moderate | Moderate | Moderate | Moderate | Yes | Yes | Unclear | Yes | Unclear | Unclear | Unclear | Yes | Unclear | Moderate |
| Oda 2020 | High | High | Moderate | Moderate | High | No | No | Unclear | Yes | No | Unclear | Unclear | Yes | No | High |
| Pimenta 2025 | High | Moderate | Low | Moderate | High | No | No | Unclear | Yes | Unclear | Yes | Unclear | Yes | Unclear | High |
| Porcari 2021 | High | Low | Low | Low | Moderate (selection) | No | No | Unclear | Yes | Yes | Yes | Yes | Yes | Yes | High |
| Quarta 2015 |  |  | Low | Low | Not directly applicable | Unclear | Unclear | Unclear | Yes | Unclear | Yes | Yes | Yes | Unclear | Moderate |
| Remior-Perez 2025 | Moderate | Moderate | Low | Moderate | Moderate | Unclear | Unclear | Unclear | Yes | Unclear | Yes | Unclear | Yes | Unclear | Moderate |
| Rosenblum 2021 | Moderate | Low | Low | Low | Low–Moderate | Unclear | Unclear | Unclear | Yes | Yes | Yes | Yes | Yes | Yes | Moderate |
| Ruiz-Hueso 2023 | Moderate | Moderate | Low | Moderate | Moderate | Unclear | Unclear | Unclear | Yes | Unclear | Yes | Unclear | Yes | Unclear | Moderate |
| SadrAldin 2024 | Moderate–High | Moderate | Moderate | Moderate | Moderate–High | No | No | Unclear | Yes | Unclear | Unclear | Unclear | Yes | Unclear | High |
| Salvalaggio 2022 | High | Moderate | Moderate–High | Moderate | High | No | No | Unclear | Yes | Unclear | No | Unclear | Unclear | Unclear | High |
| Samuelsson 2018 | Moderate | Moderate | Low | Moderate | Moderate | Unclear | Unclear | Unclear | Yes | Unclear | Yes | Unclear | Yes | Unclear | Moderate |
| Scully 2020 | Moderate | Low | Low | Low | Low–Moderate | Unclear | Unclear | Unclear | Yes | Yes | Yes | Yes | Yes | Yes | Moderate |
| Shimoni 2021 | Moderate–High | Moderate | Low | Moderate | Moderate–High | No | No | Unclear | Yes | Unclear | Yes | Unclear | Yes | Unclear | High |
| Shinzato 2025 |  |  |  |  | Moderate (atrial amyloid); indirect to CA | No | Unclear | Unclear | Yes | Yes | Yes | Yes | Yes | Yes | Moderate |
| Singal 2021 | High | High | Low | Moderate | High | No | No | Unclear | Yes | No | Yes | Unclear | Yes | No | High |
| Son 2024 | Moderate | High | Moderate–High | Moderate | Moderate–High | Unclear | Unclear | Unclear | Yes | No | No | Unclear | Unclear | No | High |
| Spaccavento 2024 |  |  |  |  | Moderate | Yes | Yes | Unclear | Yes | Unclear | Yes | Yes | Yes | Yes | Low–Moderate |
| Sperry 2018 | Moderate | Low | Low | Low | Moderate (tissue); Indirect for CA | Unclear | Unclear | Unclear | Yes | Yes | Yes | Yes | Yes | Yes | Moderate |
| Sperry 2021 | Moderate–High | Moderate | Low | Low | Moderate–High (for CA) | No | No | Unclear | Yes | Unclear | Yes | Yes | Yes | Unclear | High |
| Sugiura 2021 | Moderate | High | Low–Moderate | Low | Moderate–High (for CA); Moderate (tissue) | Unclear | Yes | Unclear | Yes | No | Yes | Yes | Yes | No | High |
| Suomalainen 2022 | Low–Moderate | Moderate | Moderate | Moderate | Moderate | Yes | Yes | Unclear | Yes | Unclear | Unclear | Unclear | Yes | Unclear | Moderate |
| Takashio 2023 | Moderate | High | Low | Moderate | Moderate–High | Unclear | Unclear | Unclear | Yes | No | Yes | Unclear | Yes | No | High |
| Tebbe 2016 | High | Low | Low | Low | High | No | No | Unclear | Yes | Yes | Yes | Yes | Yes | Yes | High |
| Ton 2017 | High | Low | Low | Low | High | No | No | Unclear | Yes | Yes | Yes | Yes | Yes | Yes | High |
| Treibel 2016 | Moderate | Low | Low | Low | Low–Moderate | Unclear | Unclear | Unclear | Yes | Yes | Yes | Yes | Yes | Yes | Moderate |
| Tubben 2024 | Moderate | Moderate | Low | Low–Moderate | Moderate | Unclear | Unclear | Unclear | Yes | Unclear | Yes | Yes | Yes | Unclear | Moderate |
| Vianello 2021 | Moderate | Moderate–High | Low | Moderate | Moderate | Unclear | Unclear | Unclear | Yes | No | Yes | Unclear | Yes | No | High |
| Westin 2022 | Moderate | Moderate | Low | Low | Low–Moderate | Unclear | Unclear | Unclear | Yes | Unclear | Yes | Yes | Yes | Unclear | Moderate |
| Yamada 2024 | Moderate–High | High | Low–Moderate | Low | Moderate–High (for CA); Moderate (tissue) | No | No | Unclear | Yes | No | Yes | Yes | Yes | No | High |
| Yamasaki 2024 | High | High | Low (verified subset) | Moderate | Moderate–High | No | No | Unclear | Yes | No | Yes | Unclear | Yes | No | High |
| Yun 2024 | Moderate–High | Moderate | Low | Low–Moderate | Moderate | No | No | Unclear | Yes | Unclear | Yes | Yes | Yes | Unclear | High |
| Zegri-Reiriz 2019 | Moderate | High | Low–Moderate | Moderate | High (overall CA); Moderate (verified subgroups) | Unclear | Unclear | Unclear | Yes | No | Yes | Unclear | Yes | No | High |
| Zhou 2025 |  |  |  |  | Moderate–High | No | Unclear | No | Yes | No | Yes | No | Yes | Unclear | High |

**Supplementary Table 10. Publication bias assessment.**

| **Setting** | **k (whole cohort)** | **Egger intercept** | **Egger SE** | **Egger t** | **Egger df** | **Egger p** | **Begg Kendall tau** | **Begg p** |
| --- | --- | --- | --- | --- | --- | --- | --- | --- |
| Whole cohort | | | | | | | | |
| HF | 20 | -10,2515 | 1,226901 | -8,35562 | 18 | 1,31E-07 | -0,41053 | 0,011101 |
| Aortic stenosis | 15 | -11,907 | 1,39588 | -8,5301 | 13 | 1,1E-06 | -0,18095 | 0,37945 |
| Orthopedic disease | 11 | -10,0544 | 1,997592 | -5,03328 | 9 | 0,000706 | -0,2 | 0,445382 |
| Further testing for CA | | | | | | | | |
| HF | 20 | -10,2515 | 1,226901 | -8,35562 | 18 | 1,31E-07 | -0,41053 | 0,011101 |
| Aortic stenosis | 13 | -11,1118 | 1,069981 | -10,3851 | 11 | 5,06E-07 | 0,102564 | 0,675433 |
| Orthopedic disease | 10 | -9,57294 | 2,795289 | -3,42467 | 8 | 0,009026 | 0,155556 | 0,600654 |

CA, cardiac amyloidosis; HF, heart failure.

**Supplementary Figure 1. Prevalence of cardiac amyloidosis in heart failure studies (whole cohorts): Forest plot.**

**Supplementary Figure 2. Prevalence of cardiac amyloidosis in aortic stenosis studies (whole cohorts): Forest plot.**

**Supplementary Figure 3. Prevalence of cardiac amyloidosis in studies on subjects with orthopedic disease (whole cohorts): Forest plot.**

**Supplementary Figure 4. Prevalence of cardiac amyloidosis in bone scintigraphy studies (whole cohorts): Forest plot.**

**Supplementary Figure 5. Prevalence of cardiac amyloidosis in left ventricular hypertrophy/hypertrophic cardiomyopathy studies (whole cohorts): Forest plot.**

**Supplementary Figure 6. Prevalence of cardiac amyloidosis in atrial fibrillation studies (whole cohorts): Forest plot.**

**Supplementary Figure 7. Prevalence of cardiac amyloidosis in studies on subjects without specific red flags (whole cohorts): Forest plot.**

**Supplementary Figure 8. Prevalence of cardiac amyloidosis in studies on subjects with peripheral neuropathy (whole cohorts): Forest plot.**

**Supplementary Figure 9. Prevalence of cardiac amyloidosis in heart failure studies (subgroups undergoing further testing): Forest plot.**

**Supplementary Figure 10. Prevalence of cardiac amyloidosis in aortic stenosis studies (subgroups undergoing further testing): Forest plot.**

**Supplementary Figure 11. Prevalence of cardiac amyloidosis in studies on subjects with orthopedic disease (subgroups undergoing further testing): Forest plot.**

**Supplementary Figure 12. Prevalence of cardiac amyloidosis in bone scintigraphy studies (subgroups undergoing further testing): Forest plot.**

**Supplementary Figure 13. Prevalence of cardiac amyloidosis in left ventricular hypertrophy/hypertrophic cardiomyopathy studies (subgroups undergoing further testing): Forest plot.**

**Supplementary Figure 14. Prevalence of cardiac amyloidosis in atrial fibrillation studies (subgroups undergoing further testing): Forest plot.**

**Supplementary Figure 15. Prevalence of cardiac amyloidosis in studies on subjects without specific red flags (subgroups undergoing further testing): Forest plot.**

**Supplementary Figure 16. Prevalence of cardiac amyloidosis in studies on subjects with peripheral neuropathy (subgroups undergoing further testing): Forest plot.**

| **Section and Topic** | **Item #** | **Checklist item** | **Location where item is reported** |
| --- | --- | --- | --- |
| **TITLE** | | |  |
| Title | 1 | Identify the report as a systematic review. | Title and Abstract |
| **ABSTRACT** | | |  |
| Abstract | 2 | See the PRISMA 2020 for Abstracts checklist. | Abstract |
| **INTRODUCTION** | | |  |
| Rationale | 3 | Describe the rationale for the review in the context of existing knowledge. | Introduction/Background |
| Objectives | 4 | Provide an explicit statement of the objective(s) or question(s) the review addresses. | End of Introduction; also Abstract aims |
| **METHODS** | | |  |
| Eligibility criteria | 5 | Specify the inclusion and exclusion criteria for the review and how studies were grouped for the syntheses. | Methods |
| Information sources | 6 | Specify all databases, registers, websites, organisations, reference lists and other sources searched or consulted to identify studies. Specify the date when each source was last searched or consulted. | Methods |
| Search strategy | 7 | Present the full search strategies for all databases, registers and websites, including any filters and limits used. | Supplementary material |
| Selection process | 8 | Specify the methods used to decide whether a study met the inclusion criteria of the review, including how many reviewers screened each record and each report retrieved, whether they worked independently, and if applicable, details of automation tools used in the process. | Methods |
| Data collection process | 9 | Specify the methods used to collect data from reports, including how many reviewers collected data from each report, whether they worked independently, any processes for obtaining or confirming data from study investigators, and if applicable, details of automation tools used in the process. | Methods |
| Data items | 10a | List and define all outcomes for which data were sought. Specify whether all results that were compatible with each outcome domain in each study were sought (e.g. for all measures, time points, analyses), and if not, the methods used to decide which results to collect. | Methods |
|  | 10b | List and define all other variables for which data were sought (e.g. participant and intervention characteristics, funding sources). Describe any assumptions made about any missing or unclear information. | Methods |
| Study risk of bias assessment | 11 | Specify the methods used to assess risk of bias in the included studies, including details of the tool(s) used, how many reviewers assessed each study and whether they worked independently, and if applicable, details of automation tools used in the process. | Methods |
| Effect measures | 12 | Specify for each outcome the effect measure(s) (e.g. risk ratio, mean difference) used in the synthesis or presentation of results. | Methods |
| Synthesis methods | 13a | Describe the processes used to decide which studies were eligible for each synthesis (e.g. tabulating the study intervention characteristics and comparing against the planned groups for each synthesis (item #5)). | Methods |
|  | 13b | Describe any methods required to prepare the data for presentation or synthesis, such as handling of missing summary statistics, or data conversions. | Methods |
|  | 13c | Describe any methods used to tabulate or visually display results of individual studies and syntheses. | Methods |
|  | 13d | Describe any methods used to synthesize results and provide a rationale for the choice(s). If meta-analysis was performed, describe the model(s), method(s) to identify the presence and extent of statistical heterogeneity, and software package(s) used. | Methods |
|  | 13e | Describe any methods used to explore possible causes of heterogeneity among study results (e.g. subgroup analysis, meta-regression). | Methods |
|  | 13f | Describe any sensitivity analyses conducted to assess robustness of the synthesized results. | Methods |
| Reporting bias assessment | 14 | Describe any methods used to assess risk of bias due to missing results in a synthesis (arising from reporting biases). | Methods |
| Certainty assessment | 15 | Describe any methods used to assess certainty (or confidence) in the body of evidence for an outcome. | Methods |
| **RESULTS** | | |  |
| Study selection | 16a | Describe the results of the search and selection process, from the number of records identified in the search to the number of studies included in the review, ideally using a flow diagram. | Figure 1; Forest plots; tables in Supplementary material |
|  | 16b | Cite studies that might appear to meet the inclusion criteria, but which were excluded, and explain why they were excluded. | Figure 1 |
| Study characteristics | 17 | Cite each included study and present its characteristics. | Results |
| Risk of bias in studies | 18 | Present assessments of risk of bias for each included study. | Results |
| Results of individual studies | 19 | For all outcomes, present, for each study: (a) summary statistics for each group (where appropriate) and (b) an effect estimate and its precision (e.g. confidence/credible interval), ideally using structured tables or plots. | Results |
| Results of syntheses | 20a | For each synthesis, briefly summarise the characteristics and risk of bias among contributing studies. | Results |
|  | 20b | Present results of all statistical syntheses conducted. If meta-analysis was done, present for each the summary estimate and its precision (e.g. confidence/credible interval) and measures of statistical heterogeneity. If comparing groups, describe the direction of the effect. | Results |
|  | 20c | Present results of all investigations of possible causes of heterogeneity among study results. | Results |
|  | 20d | Present results of all sensitivity analyses conducted to assess the robustness of the synthesized results. | Results |
| Reporting biases | 21 | Present assessments of risk of bias due to missing results (arising from reporting biases) for each synthesis assessed. | Results |
| Certainty of evidence | 22 | Present assessments of certainty (or confidence) in the body of evidence for each outcome assessed. | Results |
| **DISCUSSION** | | |  |
| Discussion | 23a | Provide a general interpretation of the results in the context of other evidence. | Discussion |
|  | 23b | Discuss any limitations of the evidence included in the review. | Discussion |
|  | 23c | Discuss any limitations of the review processes used. | Discussion |
|  | 23d | Discuss implications of the results for practice, policy, and future research. | Discussion |
| **OTHER INFORMATION** | | |  |
| Registration and protocol | 24a | Provide registration information for the review, including register name and registration number, or state that the review was not registered. | Methods |
|  | 24b | Indicate where the review protocol can be accessed, or state that a protocol was not prepared. |  |
|  | 24c | Describe and explain any amendments to information provided at registration or in the protocol. | Methods |
| Support | 25 | Describe sources of financial or non-financial support for the review, and the role of the funders or sponsors in the review. | Methods |
| Competing interests | 26 | Declare any competing interests of review authors. | Methods |
| Availability of data, code and other materials | 27 | Report which of the following are publicly available and where they can be found: template data collection forms; data extracted from included studies; data used for all analyses; analytic code; any other materials used in the review. | Methods |

**References (Supplementary material)**

1.	Aimo A, Vergaro G, Pastore MC et al. High prevalence of wild-type transthyretin cardiac amyloidosis in older adults with carpal tunnel syndrome, heart failure or increased left ventricular mass: The CAPTURE study. Eur J Heart Fail 2025.

2.	<https://jbi.global/critical-appraisal-tools>.

3.	Mohammed SF, Mirzoyev SA, Edwards WD et al. Left ventricular amyloid deposition in patients with heart failure and preserved ejection fraction. JACC Heart Fail 2014;2:113-22.

4.	González-López E, Gallego-Delgado M, Guzzo-Merello G et al. Wild-type transthyretin amyloidosis as a cause of heart failure with preserved ejection fraction. Eur Heart J 2015;36:2585-94.

5.	Bennani Smires Y, Victor G, Ribes D et al. Pilot study for left ventricular imaging phenotype of patients over 65 years old with heart failure and preserved ejection fraction: the high prevalence of amyloid cardiomyopathy. Int J Cardiovasc Imaging 2016;32:1403-1413.

6.	Arvanitis M, Chan GG, Jacobson DR, Berk JL, Connors LH, Ruberg FL. Prevalence of mutant ATTR cardiac amyloidosis in elderly African Americans with heart failure. Amyloid 2017;24:253-255.

7.	Ton VK, Bhonsale A, Gilotra NA et al. Baseline Characteristics Predict the Presence of Amyloid on Endomyocardial Biopsy. J Card Fail 2017;23:340-344.

8.	López-Sainz Á, de Haro-Del Moral FJ, Dominguez F et al. Prevalence of cardiac amyloidosis among elderly patients with systolic heart failure or conduction disorders. Amyloid 2019;26:156-163.

9.	Hahn VS, Yanek LR, Vaishnav J et al. Endomyocardial Biopsy Characterization of Heart Failure With Preserved Ejection Fraction and Prevalence of Cardiac Amyloidosis. JACC Heart Fail 2020;8:712-724.

10.	AbouEzzeddine OF, Davies DR, Scott CG et al. Prevalence of Transthyretin Amyloid Cardiomyopathy in Heart Failure With Preserved Ejection Fraction. JAMA Cardiol 2021;6:1267-1274.

11.	Devesa A, Camblor Blasco A, Pello Lázaro AM et al. Prevalence of transthyretin amyloidosis in patients with heart failure and no left ventricular hypertrophy. ESC Heart Fail 2021;8:2856-2865.

12.	Goland S, Volodarsky I, Fabricant Y et al. Wild-type TTR amyloidosis among patients with unexplained heart failure and systolic LV dysfunction. PLoS One 2021;16:e0254104.

13.	Lindmark K, Pilebro B, Sundström T, Lindqvist P. Prevalence of wild type transtyrethin cardiac amyloidosis in a heart failure clinic. ESC Heart Fail 2021;8:745-749.

14.	Jaramillo-Hidalgo J, Quezada-Feijoó M, Ramos M, Toro R, Gómez-Pavón J, Ayala-Muñoz R. Prevalence of transthyretin cardiac amyloidosis in elderly patients diagnosed with heart failure. Rev Esp Geriatr Gerontol 2023;58:8-14.

15.	Madhani A, Sabogal N, Massillon D et al. Clinical Penetrance of the Transthyretin V122I Variant in Older Black Patients With Heart Failure: The SCAN-MP (Screening for Cardiac Amyloidosis With Nuclear Imaging in Minority Populations) Study. J Am Heart Assoc 2023;12:e028973.

16.	Ruiz-Hueso R, Salamanca-Bautista P, Quesada-Simón MA et al. Estimating the Prevalence of Cardiac Amyloidosis in Old Patients with Heart Failure-Barriers and Opportunities for Improvement: The PREVAMIC Study. J Clin Med 2023;12.

17.	Spaccavento A, Rodríguez MDR, Meretta A et al. Prevalence of transthyretin amyloid cardiomyopathy in patients admitted for acute heart failure. Curr Probl Cardiol 2024;49:102385.

18.	Tubben A, Tingen HSA, Prakken NHJ et al. Prevalence of wild-type transthyretin amyloidosis in a prospective heart failure cohort with preserved and mildly reduced ejection fraction: Results of the Amylo-VIP-HF study. Eur J Heart Fail 2024;26:695-698.

19.	Yun S, Palladini G, Anderson LJ et al. International prevalence of transthyretin amyloid cardiomyopathy in high-risk patients with heart failure and preserved or mildly reduced ejection fraction. Amyloid 2024;31:291-301.

20.	Achten A, van Empel VPM, Weerts J et al. Prevalence of transthyretin amyloid cardiomyopathy in an unselected cohort with heart failure with preserved ejection fraction. Neth Heart J 2025;33:193-198.

21.	García-Pavía P, García-Pinilla JM, Lozano-Bahamonde A et al. Prevalence of transthyretin cardiac amyloidosis in patients with heart failure with preserved ejection fraction: the PRACTICA study. Rev Esp Cardiol (Engl Ed) 2025;78:301-310.

22.	Healy L, Giblin G, Gray A et al. Prevalence of transthyretin cardiac amyloidosis in undifferentiated heart failure with preserved ejection fraction. ESC Heart Fail 2025;12:1176-1182.

23.	Nietlispach F, Webb JG, Ye J et al. Pathology of transcatheter valve therapy. JACC Cardiovasc Interv 2012;5:582-590.

24.	Longhi S, Lorenzini M, Gagliardi C et al. Coexistence of Degenerative Aortic Stenosis and Wild-Type Transthyretin-Related Cardiac Amyloidosis. JACC Cardiovasc Imaging 2016;9:325-7.

25.	Treibel TA, Fontana M, Gilbertson JA et al. Occult Transthyretin Cardiac Amyloid in Severe Calcific Aortic Stenosis: Prevalence and Prognosis in Patients Undergoing Surgical Aortic Valve Replacement. Circ Cardiovasc Imaging 2016;9.

26.	Castaño A, Narotsky DL, Hamid N et al. Unveiling transthyretin cardiac amyloidosis and its predictors among elderly patients with severe aortic stenosis undergoing transcatheter aortic valve replacement. Eur Heart J 2017;38:2879-2887.

27.	Cavalcante JL, Rijal S, Abdelkarim I et al. Cardiac amyloidosis is prevalent in older patients with aortic stenosis and carries worse prognosis. J Cardiovasc Magn Reson 2017;19:98.

28.	Nitsche C, Aschauer S, Kammerlander AA et al. Light-chain and transthyretin cardiac amyloidosis in severe aortic stenosis: prevalence, screening possibilities, and outcome. Eur J Heart Fail 2020;22:1852-1862.

29.	Oda S, Kidoh M, Takashio S et al. Quantification of Myocardial Extracellular Volume With Planning Computed Tomography for Transcatheter Aortic Valve Replacement to Identify Occult Cardiac Amyloidosis in Patients With Severe Aortic Stenosis. Circ Cardiovasc Imaging 2020;13:e010358.

30.	Scully PR, Patel KP, Treibel TA et al. Prevalence and outcome of dual aortic stenosis and cardiac amyloid pathology in patients referred for transcatheter aortic valve implantation. Eur Heart J 2020;41:2759-2767.

31.	Nitsche C, Scully PR, Patel KP et al. Prevalence and Outcomes of Concomitant Aortic Stenosis and Cardiac Amyloidosis. J Am Coll Cardiol 2021;77:128-139.

32.	Rosenblum H, Masri A, Narotsky DL et al. Unveiling outcomes in coexisting severe aortic stenosis and transthyretin cardiac amyloidosis. Eur J Heart Fail 2021;23:250-258.

33.	Shimoni S, Zikri M, Haberman D et al. Transthyretin cardiac amyloidosis in patients after TAVR: clinical and echocardiographic findings and long term survival. ESC Heart Fail 2021;8:4549-4561.

34.	Singal AK, Bansal R, Singh A et al. Concomitant Transthyretin Amyloidosis and Severe Aortic Stenosis in Elderly Indian Population: A Pilot Study. JACC CardioOncol 2021;3:565-576.

35.	Costa J, El-Ali A, Morland D et al. Cardiac amyloidosis prevalence and 1-year outcome in patients with aortic stenosis undergoing transaortic valve implantation: Findings from the CAMPOS-TAVI study. Arch Cardiovasc Dis 2024;117:461-469.

36.	Jakstaite AM, Vogel JK, Luedike P et al. Screening for Occult Transthyretin Amyloidosis in Patients with Severe Aortic Stenosis and Amyloid Red Flags. J Clin Med 2024;13.

37.	SadrAldin R, Ahmed J, Alkaf F et al. Prevalence of transthyretin cardiac amyloidosis in patients with aortic stenosis. Am J Cardiovasc Dis 2024;14:384-395.

38.	McShane JN, Tavoosi A, El Mais H et al. CT derived ECV in severe aortic stenosis: prognosticator and screening test for co-existent transthyretin cardiac amyloidosis. Am Heart J Plus 2025;56:100575.

39.	Sperry BW, Reyes BA, Ikram A et al. Tenosynovial and Cardiac Amyloidosis in Patients Undergoing Carpal Tunnel Release. J Am Coll Cardiol 2018;72:2040-2050.

40.	Zegri-Reiriz I, de Haro-Del Moral FJ, Dominguez F et al. Prevalence of Cardiac Amyloidosis in Patients with Carpal Tunnel Syndrome. J Cardiovasc Transl Res 2019;12:507-513.

41.	Sperry BW, Khedraki R, Gabrovsek A et al. Cardiac Amyloidosis Screening at Trigger Finger Release Surgery. Am J Cardiol 2021;160:96-98.

42.	Sugiura K, Kozuki H, Ueba H et al. Tenosynovial and Cardiac Transthyretin Amyloidosis in Japanese Patients Undergoing Carpal Tunnel Release. Circ Rep 2021;3:338-344.

43.	Vianello PF, La Malfa G, Tini G et al. Prevalence of transthyretin amyloid cardiomyopathy in male patients who underwent bilateral carpal tunnel surgery: The ACTUAL study. Int J Cardiol 2021;329:144-147.

44.	Baylor JL, Nester JR, Olsen HP, Pallis M, Akoon A, Grandizio LC. Prevalence of Amyloid Deposition in Patients Undergoing Surgical Repair of Traumatic Distal Biceps Tendon Ruptures. J Hand Surg Glob Online 2022;4:344-347.

45.	Westin O, Fosbøl EL, Maurer MS et al. Screening for Cardiac Amyloidosis 5 to 15 Years After Surgery for Bilateral Carpal Tunnel Syndrome. J Am Coll Cardiol 2022;80:967-977.

46.	Debonnaire P, Claeys M, De Paepe P et al. Prospective Screening for Transthyretin Cardiac Amyloidosis in Spinal Stenosis Surgery Patients: Results of the CASS Study. JACC CardioOncol 2023;5:836-838.

47.	Takashio S, Kato T, Tashima H et al. Prevalence of Cardiac Amyloidosis in Patients Undergoing Carpal Tunnel Release With Amyloid Deposition. Circ J 2023;87:1047-1055.

48.	Andrei V, Argirò A, Mazzoni C et al. Screening for cardiac amyloidosis in patients with tenosynovial red flags: A collaboration between family medicine and cardiology. Int J Cardiol 2024;407:132114.

49.	Gannon NP, Ward CM. Results of Implementation of Amyloidosis Screening for Patients Undergoing Carpal Tunnel Release. J Hand Surg Am 2024;49:675-680.

50.	Yamada E, Umemoto T, Taguchi T et al. Prevalence of amyloid deposition and cardiac amyloidosis in shoulder disease compared to carpal tunnel syndrome. JSES Int 2024;8:349-354.

51.	Eldhagen P, Tzortzakakis A, Lund LH et al. Cardiac amyloidosis after lumbar spinal stenosis surgery - a comprehensive prospective cohort study. Amyloid 2025:1-8.

52.	Pimenta S, Santos L, Martins A et al. Screening for Cardiac Amyloidosis When Conducting Carpal Tunnel Surgery. J Clin Med 2025;14.

53.	al-Nahhas AM, Jinnouchi S, Anagnostopoulos C, Hirsch W, Heary T, McCready VR. Clinical significance of technetium-99m methylene diphosphonate myocardial uptake: association with carcinoma of the prostate. Eur J Nucl Med 1995;22:148-53.

54.	Longhi S, Guidalotti PL, Quarta CC et al. Identification of TTR-related subclinical amyloidosis with 99mTc-DPD scintigraphy. JACC Cardiovasc Imaging 2014;7:531-2.

55.	Mohamed-Salem L, Santos-Mateo JJ, Sanchez-Serna J et al. Prevalence of wild type ATTR assessed as myocardial uptake in bone scan in the elderly population. Int J Cardiol 2018;270:192-196.

56.	Kim HM, Sohn DW, Paeng JC. Prevalence of Positive (99 m)Tc-DPD Scintigraphy as an Indicator of the Prevalence of Wild-type Transthyretin Amyloidosis in the Elderly. Int Heart J 2019;60:643-647.

57.	Bianco M, Parente A, Biolè C et al. The prevalence of TTR cardiac amyloidosis among patients undergoing bone scintigraphy. J Nucl Cardiol 2021;28:825-830.

58.	Cuscaden C, Ramsay SC, Prasad S, Goodwin B, Smith J. Estimation of prevalence of transthyretin (ATTR) cardiac amyloidosis in an Australian subpopulation using bone scans with echocardiography and clinical correlation. J Nucl Cardiol 2021;28:2845-2856.

59.	Halme HL, Ihalainen T, Suomalainen O et al. Convolutional neural networks for detection of transthyretin amyloidosis in 2D scintigraphy images. EJNMMI Res 2022;12:27.

60.	Nitsche C, Mascherbauer K, Calabretta R et al. Prevalence and Outcomes of Cardiac Amyloidosis in All-Comer Referrals for Bone Scintigraphy. J Nucl Med 2022;63:1906-1911.

61.	Salvalaggio A, Cipriani A, Righetto S et al. Incidental cardiac uptake of (99m)Tc-diphosphonates is predictive of poor outcome: data from 9616 bone scintigraphies. J Nucl Cardiol 2022;29:3419-3425.

62.	Suomalainen O, Pilv J, Loimaala A, Mätzke S, Heliö T, Uusitalo V. Prognostic significance of incidental suspected transthyretin amyloidosis on routine bone scintigraphy. J Nucl Cardiol 2022;29:1021-1029.

63.	Navarro-Saez MDC, Feijoo-Massó C, Bravo Ferrer ZDC et al. Trends in diagnosis of cardiac transthyretin amyloidosis: 3-year analysis of scintigraphic studies: Prevalence of myocardial uptake and its predictor factors. Int J Cardiovasc Imaging 2023;39:1397-1404.

64.	Son J, Han YH, Lee SH. Prevalence and characteristics of patients with incidental cardiac uptake on bone scintigraphy. J Cardiovasc Imaging 2024;32:19.

65.	Cariou E, Bennani Smires Y, Victor G et al. Diagnostic score for the detection of cardiac amyloidosis in patients with left ventricular hypertrophy and impact on prognosis. Amyloid 2017;24:101-109.

66.	Maurizi N, Rella V, Fumagalli C et al. Prevalence of cardiac amyloidosis among adult patients referred to tertiary centres with an initial diagnosis of hypertrophic cardiomyopathy. Int J Cardiol 2020;300:191-195.

67.	Nakao Y, Saito M, Inoue K et al. Cardiac amyloidosis screening using a relative apical sparing pattern in patients with left ventricular hypertrophy. Cardiovasc Ultrasound 2021;19:30.

68.	Merlo M, Pagura L, Porcari A et al. Unmasking the prevalence of amyloid cardiomyopathy in the real world: results from Phase 2 of the AC-TIVE study, an Italian nationwide survey. Eur J Heart Fail 2022;24:1377-1386.

69.	Remior-Pérez P, Gómez-Molina M, García-Rodríguez D et al. Prevalence of Cardiac Amyloidosis Among Elderly Patients With Recent-Onset Atrial Fibrillation: The PREVAL-ATTR Study. Can J Cardiol 2025;41:167-177.

70.	Yamasaki H, Kondo H, Shiroo T et al. Efficacy of Computed Tomography-Based Evaluation of Myocardial Extracellular Volume Combined With Red Flags for Early Screening of Concealed Cardiac Amyloidosis in Patients With Atrial Fibrillation. Circ J 2024;88:1167-1175.

71.	Shinzato K, Takahashi Y, Yamaguchi T et al. Atrial amyloidosis identified by biopsy in atrial fibrillation: prevalence and clinical presentation. Eur Heart J 2025.

72.	Porcari A, Bussani R, Merlo M et al. Incidence and Characterization of Concealed Cardiac Amyloidosis Among Unselected Elderly Patients Undergoing Post-mortem Examination. Front Cardiovasc Med 2021;8:749523.

73.	Aimo A, Sanguinetti C, Franzini M. Screening for Cardiac Involvement in Carriers of Pathogenic TTR Variants: Proposal for an Approach Based on High-Sensitivity Troponin. Circulation 2024;150:1656-1658.

74.	Arima N, Ochi Y, Kubo T et al. Prospective Multicenter Screening With High-Sensitivity Cardiac Troponin T for Wild-Type Transthyretin Cardiac Amyloidosis in Outpatient and Community-Based Settings. Circ J 2024;89:24-30.

75.	Di Stefano V, Lupica A, Alonge P et al. Genetic screening for hereditary transthyretin amyloidosis with polyneuropathy in western Sicily: Two years of experience in a neurological clinic. Eur J Neurol 2024;31:e16065.

76.	Doneddu PE, Moretti G, Di Stefano V et al. Prevalence of hereditary transthyretin amyloidosis in CIDP patients with red flags: a multicenter genetic screening and misdiagnosis analysis. J Neurol 2025;272:515.

77.	Samuelsson K, Radovic A, Press R et al. Screening for Fabry disease and Hereditary ATTR amyloidosis in idiopathic small-fiber and mixed neuropathy. Muscle Nerve 2019;59:354-357.

78.	de Frutos F, Caraballo Ramos I, Martínez Chaves V, Corral Azor AM, Berchíd Débdi MS, García-Pavía P. Results of a population screening program for hereditary transthyretin amyloidosis. Rev Esp Cardiol (Engl Ed) 2024;77:498-500.

79.	Di Stefano V, Messina C, Pignolo A et al. Genetic Screening for Hereditary Transthyretin Amyloidosis in the Population of Cammarata and San Giovanni Gemini Through Red Flags and Registry Archives. Brain Sci 2025;15.

80.	Cannie D, Patel K, Protonotarios A et al. Prevalence of transthyretin cardiac amyloidosis in patients with high-degree AV block. Open Heart 2024;11.

81.	Mangiacavalli S, Milani P, Cartia CS et al. Feasibility of a Biomarker-Based Screening for Pre-Symptomatic AL Amyloidosis in Patients With Intermediate/High-Risk MGUS. Am J Hematol 2025;100:342-345.

82.	Zhou P, Mansukhani MM, Yeh R et al. Screening for Systemic Light-Chain Amyloidosis in Patients Over 60 with λ Monoclonal Gammopathies. J Clin Med 2025;14.

83.	Quarta CC, Buxbaum JN, Shah AM et al. The amyloidogenic V122I transthyretin variant in elderly black Americans. N Engl J Med 2015;372:21-9.

84.	Tebbe U, Bramlage K, John F et al. Endomyocardial biopsy in patients with cardiomyopathy of unknown origin: does specialized center experience apply to a tertiary care hospital? BMC Res Notes 2016;9:459.
